# *Plasmodium falciparum* PhIL1-associated complex plays an essential role in merozoite reorientation and invasion of host erythrocytes

**DOI:** 10.1371/journal.ppat.1009750

**Published:** 2021-07-29

**Authors:** Ekta Saini, Pradeep Kumar Sheokand, Vaibhav Sharma, Prakhar Agrawal, Inderjeet Kaur, Shailja Singh, Asif Mohmmed, Pawan Malhotra

**Affiliations:** 1 International Centre for Genetic Engineering and Biotechnology, New Delhi, India; 2 Special Centre for Molecular Medicine, Jawaharlal Nehru University, New Delhi, India; Seattle Children’s Research Institute, UNITED STATES

## Abstract

The human malaria parasite, *Plasmodium falciparum* possesses unique gliding machinery referred to as the glideosome that powers its entry into the insect and vertebrate hosts. Several parasite proteins including Photosensitized INA-labelled protein 1 (PhIL1) have been shown to associate with glideosome machinery. Here we describe a novel PhIL1 associated protein complex that co-exists with the glideosome motor complex in the inner membrane complex of the merozoite. Using an experimental genetics approach, we characterized the role(s) of three proteins associated with PhIL1: a glideosome associated protein- PfGAPM2, an IMC structural protein- PfALV5, and an uncharacterized protein—referred here as PfPhIP (PhIL1 Interacting Protein). Parasites lacking PfPhIP or PfGAPM2 were unable to invade host RBCs. Additionally, the downregulation of PfPhIP resulted in significant defects in merozoite segmentation. Furthermore, the PfPhIP and PfGAPM2 depleted parasites showed abrogation of reorientation/gliding. However, initial attachment with host RBCs was not affected in these parasites. Together, the data presented here show that proteins of the PhIL1-associated complex play an important role in the orientation of *P*. *falciparum* merozoites following initial attachment, which is crucial for the formation of a tight junction and hence invasion of host erythrocytes.

## Author summary

Invasion of *Plasmodium* merozoites into RBCs is a multistep process that involves initial attachment of merozoites to the RBC surface, their reorientation, and subsequent gliding into RBCs using glideosome machinery. The glideosome machinery lies between the plasma membrane and inner membrane complex (IMC) and consists of MyoA, its interacting protein; MTIP, gliding associated proteins (GAPs), and a Photosensitized INA labeled protein (PhIL1)-associated protein complex. Here, we demonstrate that the deletion of any of two components of the PhIL1-associated complex, PfPhIP or PfGAPM2, aborts merozoite reorientation and blocks their invasion into RBCs. The study thus provides new molecular and mechanistic insights into merozoite invasion of RBCs.

## Introduction

Invasion of *Plasmodium falciparum* merozoites during the asexual blood stages is a multistep process that involves initial contact, reorientation, active invasion by gliding motility and resealing [1]. All these steps are highly regulated and are mediated through a series of interactions between distinct *Plasmodium* and host proteins [1–4]. Successful invasion of human erythrocytes is essential for parasite survival and therefore is the target of malaria vaccine and inhibitor(s) development. One of the unique steps in the invasion of apicomplexan zoites including *Plasmodium* merozoite is the gliding motility that powers parasites’ to cross non-permissive biological host membranes [5]. *P*. *falciparum* and *T*. *gondii* have unique gliding machinery powered by an actomyosin motor termed the glideosome, which brings about the gliding of zoites into the host. The glideosome is anchored to the inner membrane complex (IMC) that is composed of flattened membrane cisternae or alveolar vesicles, whose cytoplasmic faces are connected to subpellicular microtubules and a subpellicular protein network (SPN). The IMC of an *Apicomplexan* parasite plays diverse roles in maintaining the structural stability of the zoite forms. It also acts as a scaffold for daughter cell development and plays a key role in motility and host-cell invasion [6,7]. Proteins involved in the organization of pellicle/IMC/glideosome include structural proteins such as alveolins (IMC1a-h), glideosome associated protein- 40, -45, and -50 and glideosome associated proteins with multiple membrane spans (GAPMs), ISPs and these proteins together with MTIP (Myosin A tail domain interacting protein) anchor the actomyosin motor complex to the IMC [8,9]. These structures and proteins of glideosome/IMC are important as any disruption in the assembly of IMC blocks parasite invasion as well as sexual stage development [6,10].

Despite these known IMC-associated protein families and complexes, the underlying functions of IMC and its core complexes are still unexplored. We and others have shown that *Plasmodium* PhIL1 is localized to the IMC and is required for both asexual and sexual stages of parasite development [10,11]. Gene disruption of PhIL1 prevented the formation of transmittable mature gametocytes [10]. Analysis of PfPhIL1 interactome identified Alveolin (ALV5), glideosome associated proteins GAP50, GAPM1, -2 and -3 and few novel uncharacterized proteins such as PhIL1 interacting protein (PF3D7_1310700), PF3D7_1355600 (PIP1), PF3D7_1431100 and PF3D7_1430880 and PF3D7_1117000 [10,11].

In the present study, we selected three PhIL1 interacting proteins, a glideosome associated protein- PfGAPM2, an IMC structural protein- PfALV5, and a newly identified protein PF3D7_1310700- referred here as PfPhIP (PhIL1 interacting protein) and investigated the function of PhIL1-associated novel complex. Phenotypic analysis of parasites lacking PfGAPM2 and PfPhIP showed the role of these proteins in the reorientation of *P*. *falciparum* merozoites that disrupted their invasion into the human erythrocytes. The work thus identifies a novel PhIL1- associated protein complex and its role in the reorientation of merozoite towards the host surface, a step essential for tight junction formation and subsequent invasion of merozoite into RBC.

## Results

### *P*. *falciparum* ALV5, PhIP, and GAPM2 proteins co-localize with PhIL1 in the inner membrane complex

**Fig 1:**
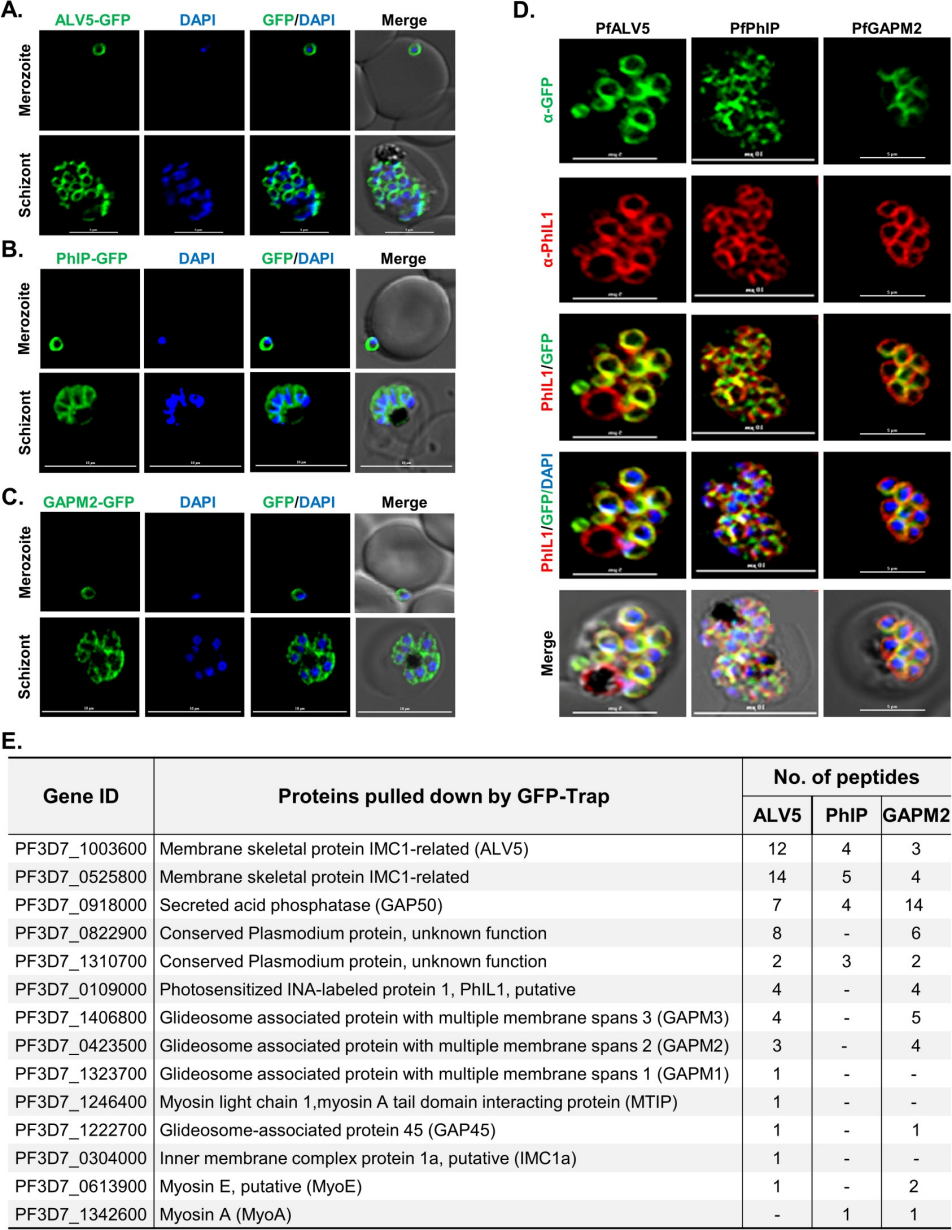
PhIL1 colocalize with ALV5, PhIP, and GAPM2 in the *P*. *falciparum* IMC. **(A)** ALV5-GFP expression pattern in asexual blood stages (merozoite, and schizont) of *P*. *falciparum*. **(B)** PhIP-GFP expression pattern in asexual blood stages showed typical IMC localization. **(C)** GAPM2-GFP expression pattern in asexual blood stages. **(D)** Co-staining of PhIL1 with ALV5, PhIP, and GAPM2 in *P*. *falciparum* blood-stage schizont. These proteins co-localized with PhIL1 in the IMC at the schizont stage of the parasite with a Pearson’s colocalization coefficient of more than 0.7. Scale bar = 5 μm. **(E)** List of proteins pulled down by GFP-trap beads from lysates obtained from parasites expressing GFP-tagged ALV5, PhIP, or GAPM2 respectively. n = 3 experiments.

To investigate the association of *P*. *falciparum* PhIL1 with other parasite proteins as shown in our previous interactome analysis [11], we expressed three proteins: a conserved protein of unknown function (PF3D7_1310700) referred here as PhIL1 Interacting Protein (PhIP), Alveolin 5 (PF3D7_1003600 or IMC1c), and GAPM2 (PF3D7_0423500) having molecular weights 16.53 kDa, 32.66 kDa, and 42.66 kDa respectively, as GFP-tagged fusion proteins in the parasite (S1A Fig). Expression of the fusion protein was confirmed by the western blot analysis of lysate from the transgenic parasites using anti-GFP antibodies (S1B, S1C, and S1D Fig). Transgenic parasites expressing ALV5-GFP showed peripheral localization in the schizont and merozoite stages (Fig 1A). Parasites expressing PhIP-GFP or GAPM2-GFP showed a similar pattern of peripheral localization in the IMC (Fig 1B and 1C). These proteins co-localized with PhIL1 in the IMC at the schizont stage of the parasite with a Pearson’s colocalization coefficient of more than 0.7 in an indirect immunofluorescence assay (Fig 1D).

Subsequently, we performed pull-down assays using GFP-Trap beads with the parasite extracts prepared from these transgenic lines. Immunoprecipitates were analyzed by mass spectrometry to identify the interacting partners. The glideosomal proteins GAP50, glideosome associated proteins with multiple membrane spans (GAPMs) 1, -2 and -3, and alveolin/ IMC protein family were identified in each precipitate, together with PhIP (PF3D7_1310700) and ALV5 (Fig 1E). Overall, these results confirmed the interactions among these proteins as well as with the PhIL1 protein.

### PhIL1 associated novel complex is closely associated with the glideosomal complex

**Fig 2:**
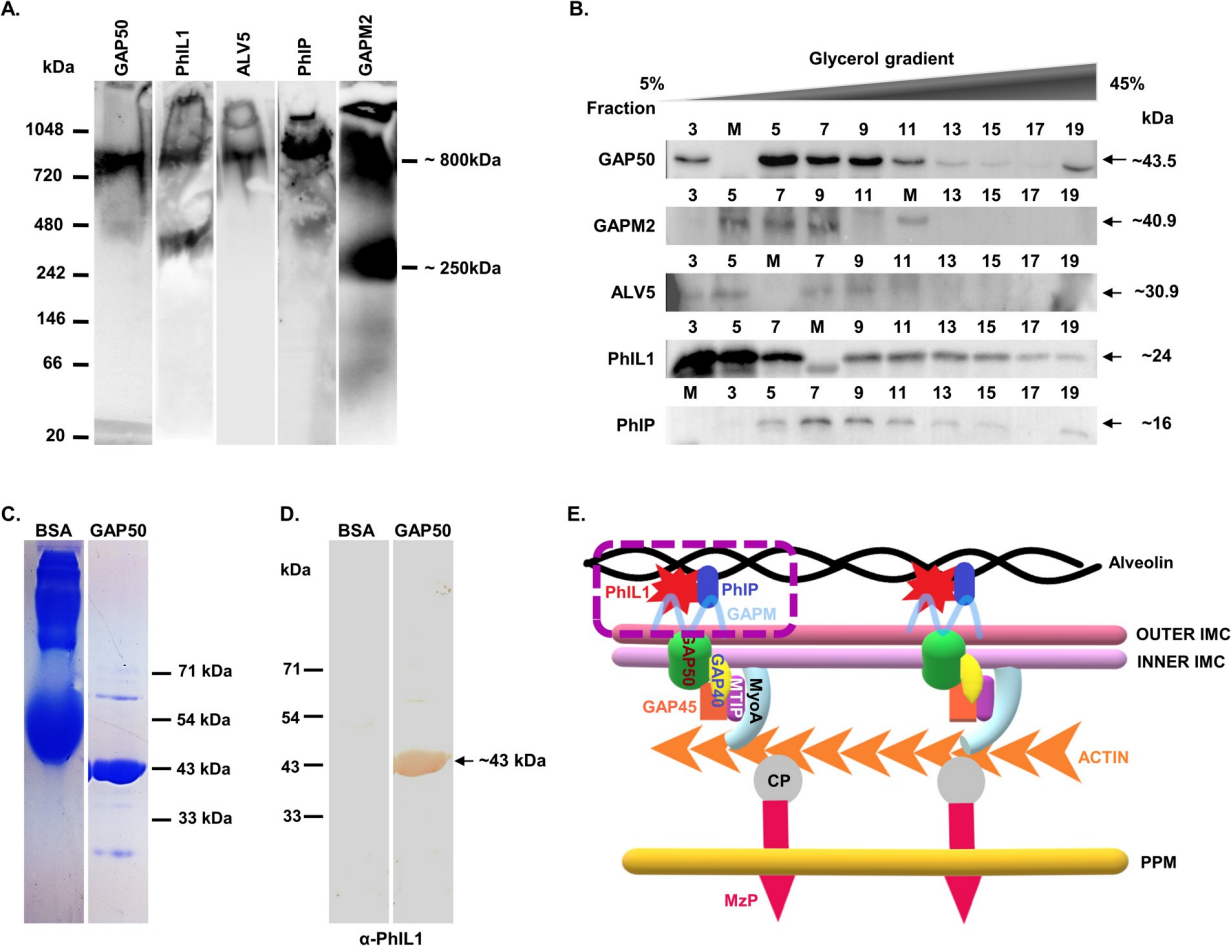
PhIL1-associated novel complex overlaps with components of the linear motor in the IMC but is a separate complex. **(A)** Native PAGE analysis of schizont stage parasite lysate shows co-existence of PhIL1-associated complex with the glideosomal complex via. some overlapping components. A complex of ∼800 kDa was detected containing components of PfPhIL1-associated complex and GAP50. Furthermore, a smaller complex of ∼250 kDa was also detected that included PfPhIL1 and PfGAPM2. These results indicate a possible association of PhIL1-associated complex and glideosomal complex. n = 2 experiments. **(B)** PhIL1-associated complex co-exists with the glideosomal complex as seen by Glycerol gradient co-sedimentation analysis. Glycerol gradient fractionation of *P*. *falciparum* schizont extract using 5 to 45% Glycerol gradient followed by immunoblotting of the fractions using specific antibodies showed the co-sedimentation of PfGAP50, PfPhIL1, PfALV5, PfPhIP, and PfGAPM2 together in fractions 5 to 11, particularly, in fraction 9 corresponding to ∼250 kDa molecular mass. n = 2 experiments. **(C)** Recombinant GAP50 and BSA were subjected to SDS-PAGE followed by **(D)** far western analysis showing the interaction of recombinant PfGAP50 protein with PfPhIL1. GAP50 was denatured and renatured on the membrane followed by incubation with recombinant PhIL1 protein and probed with α-PhIL1 antisera which recognized a band of ~43kDa corresponding to the size of GAP50. BSA used as control protein did not show any interaction with PfPhIL1. **(E)** Model showing molecular motor and partially overlying proposed PhIL1-associated complex (dotted box) in the parasite IMC. Photosensitized 5-[^125^I] Iodonaphthalene-1-azide Labelled Protein-1 (PhIL1); **Ph**IL1 **I**nteracting **P**rotein (PhIP); Glideosome-Associated Protein 40, 45 and 50 (GAP40, 45 and 50); Glideosome-associated protein with multiple membrane spans (GAPM); myosin-A (MyoA); Myosin-A Tail domain Interacting Protein (MTIP); Connector Protein between actin and protein of the merozoite surface (CP); Protein of the Merozoite surface (MzP); Parasite Plasma Membrane (PPM).

To test the existence of PhIL1-associated protein complex and its link with the glideosomal complex, if any, schizont stage parasite lysate was subjected to blue native PAGE followed by immunoblot analysis. As shown in Fig 2A, two bands, a high molecular weight band of ~800kDa and a low molecular weight complex of ~250kDa were observed, when the gel was immunoblotted with anti-PhIL1 or anti-GAPM2 antibodies (Fig 2A). In comparison, anti-GAP50 or anti-PhIP, or anti-ALV5 antibodies recognized a single band of ~800 kDa. These results indicated the involvement of PhIL1 and GAPM2 in two independent complexes that are composed of different but overlapping proteins.

To further substantiate these results, we performed sedimentation analysis of *P*. *falciparum* 3D7 schizont/ merozoite lysate using glycerol density gradient centrifugation. Western blot analysis of the glycerol gradient fractions revealed that PhIP, GAPM2, and ALV5 proteins co-sedimented together with PhIL1 in fractions 5 to 11, particularly in fraction 9 corresponding to ∼250 kDa molecular mass (Figs 2B and S2A–S2E), suggesting that these proteins probably are associated together in the parasite. *In-vitro* protein-protein interaction tool far-western blot analysis [12] provided additional evidence for the co-existence of PfPhIL1 and PfGAP50. We recombinantly expressed PfPhIL1 [11] and PfGAP50 (Fig 2C) proteins and performed far-western blotting. The far-western analysis strongly detected the interaction between membrane-bound recombinant PfGAP50 protein and soluble recombinant PfPhIL1 bait protein (Fig 2D).

Taken together, results presented here (Figs 1 and 2) validate the association of these proteins with each other and in particular, their interactions with select but not all components of the glideosome machinery. The data thus illustrates that these proteins probably form an independent complex in the IMC, which may have a diverse role than the glideosome complex described earlier. Based on the above results we propose that PhIL1 forms a novel complex probably in the outer IMC, having overlapping components with the glideosomal motility complex. The organization of the proposed novel PhIL1-associated complex is depicted in Fig 2E (boxed).

### PhIL1-associated complex plays an important role in parasite development and invasion

To address the role of PfALV5, PfPhIP, and PfGAPM2 proteins in *P*. *falciparum*, we generated conditional knock-down parasite lines expressing respective genes in fusion with HA-*glm*S. The *glm*S ribozyme is expressed downstream of the target gene, which is efficiently knocked down in response to glucosamine (GlcN). The strategy for generating the knock-down lines is presented in S3A Fig. Integration into the parasite genome was confirmed by PCR analysis (S3B, S3C, and S3D Fig).

**Fig 3:**
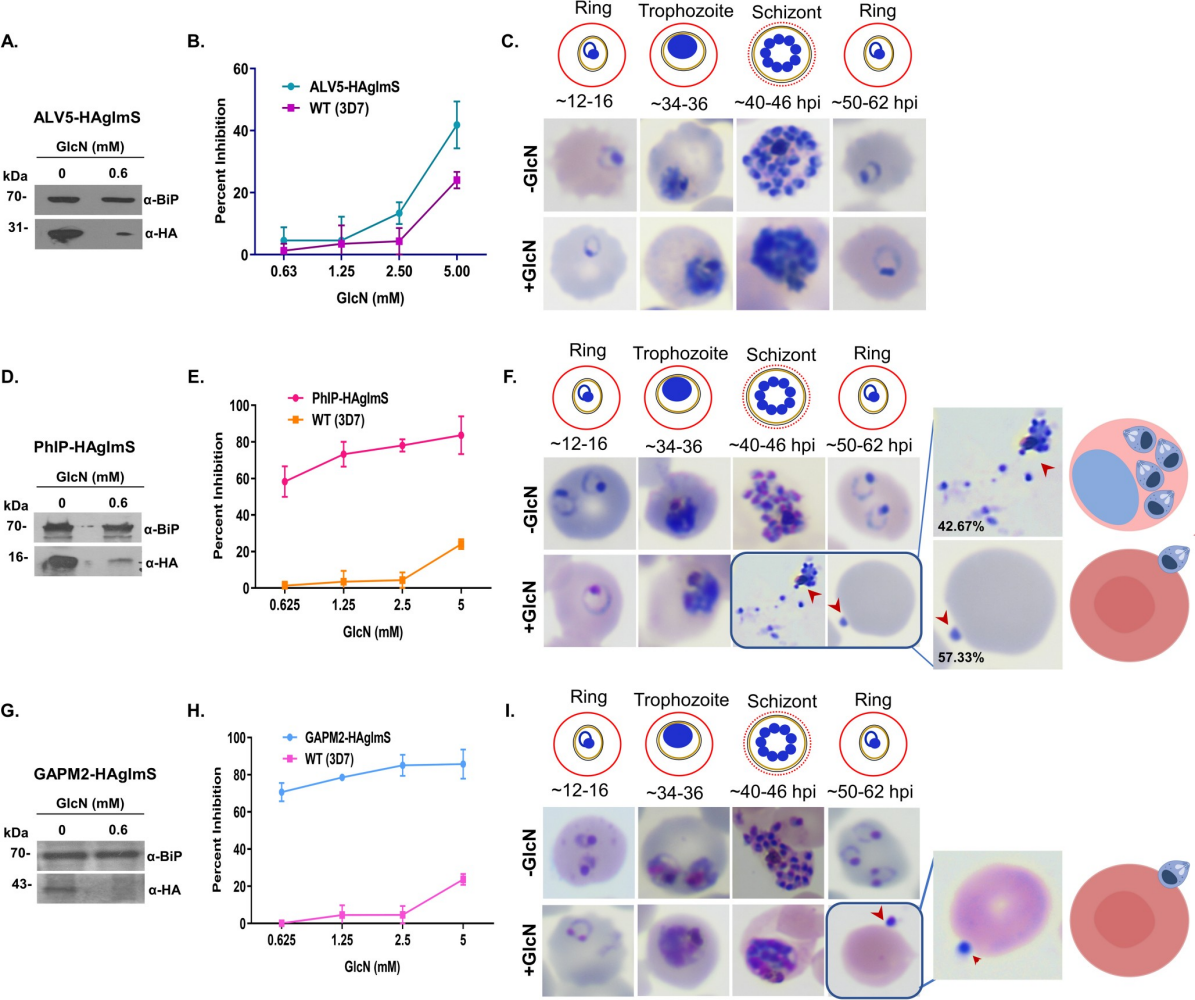
Functional characterization of *P*. *falciparum* ALV5, PhIP, and GAPM2 by inducible regulation of endogenous protein levels and their effect on parasite growth. **(A)** Western blot analysis of lysate from PfALV5-pHA-*glmS* line with α-HA rat serum showing conditional knock-down on glucosamine treatment. **(B)** Effect of conditional knockdown of PfALV5 on parasite invasion of the host RBC. Glucosamine-treated (+GlcN) and untreated (-GlcN) cultures were incubated till the formation of new rings and the parasitemia was estimated by flow cytometry. **(C)** Representative parasites from the Giemsa-stained smears showing morphology following ALV5 knockdown. **(D)** Western blot analysis of lysate from PfPhIP-HA-*glmS* line with α-HA rat antibody to check for conditional knock-down shows a robust knockdown of PhIP-HA protein. **(E)** Effect of conditional knockdown of PfPhIP on parasite invasion. **(F)** Representative parasites from the Giemsa-stained smears showing morphology following PhIP knockdown. Percentage of different phenotypes was calculated from Giemsa smears of glucosamine treated PhIP-HA-*glmS* parasites, highlighting the arrest of growth in segmented schizont stage, and altered efficacy of released merozoites to invade following PhIP knockdown. See also S4 Fig. **(G)** Western blot analysis of lysate from PfGAPM2-HA-*glmS* line with α-HA rat serum showing efficient knockdown of the GAPM2-HA protein when compared to the *Pf*BiP loading control. **(H)** Effect of conditional knockdown of PfGAPM2 in parasite showing up to 85% invasion inhibition. **(I)** Representative Giemsa-stained smears showing the arrest in development following GAPM2 knockdown due to inefficiency of released merozoites to invade the host RBC. Zoomed Giemsa smear show phenotype for PhIP- and GAPM2-HA-*glmS* parasites in presence of glucosamine, highlighting the arrest in development following knockdown. Data represent mean ± SD. n = 3 experiments. *Pf*BiP was used as a loading control for western blot analysis.

Expression and efficient knockdown of the fusion protein was analyzed by western blot of lysate from transgenic parasites using anti-HA antibody under the effect of GlcN inducer (Fig 3A, 3D and 3G). Ring stage parasites at 16–20 hpi were treated with GlcN (2.5 mM), parasites were harvested at 42–44 hpi and the lysate was subjected to SDS-PAGE. PfBiP, a constitutively expressed endoplasmic reticulum chaperone protein was used as a loading control. Apparent knockdown of up to ~80–85% was achieved for the expression of ALV5-HA, PhIP-HA, and GAPM2-HA in the respective parasite lysates.

GlcN was added at the ring stage parasites 16–20 hpi at varying concentrations (0.6 mM, 1.25 mM, 2.5 mM, and 5 mM) and the parasite growth was monitored till the formation of new rings i.e., up till one invasion cycle. Loss of ALV5 had little effect in the invasion of human RBCs by merozoites in comparison to the wild-type parasites (Fig 3B). However, PhIP depleted parasites showed ~80% invasion inhibition at 5 mM glucosamine (Fig 3E) while a reduction in GAPM2 levels exhibited an invasion inhibitory potential of ~80% at 1.25 mM concentration of GlcN (Fig 3H).

Representative Giemsa-stained smears of the ALV5 knockdown parasites showed no significant difference apart from a slightly delayed parasite growth cycle in the GlcN treated (5 mM) and untreated parasites suggesting that ALV5 is not essential for parasite growth (Fig 3C). In comparison, the PhIP-HA-*glm*S parasites showed arrested development of schizonts (1.25 mM GlcN treatment) (Fig 3F). In PhIP knockdown parasites two distinct phenotypes were observed, ~43% showed unsegmented merozoites while 57% of the PhIP depleted schizonts egressed normally however, these merozoites were unable to invade, and newly released merozoites were seen arrested on the surface of RBC suggesting that despite the initial attachment, the parasite was unable to penetrate the host RBC (Figs 3F-zoom and S4). In the absence of GlcN, distinct merozoites were observed enclosed in all schizonts and these merozoites invaded normally as seen with their ability to progress to ring stage (Fig 3F). By contrast, treatment of GAPM2-HA-*glm*S with GlcN resulted in normal merozoite egress, however released merozoites were found to be stuck at the erythrocyte surface, indicative of their inability to invade the RBC (Fig 3I).

### PfPhIP knock-down results in underdeveloped IMC during schizogony leading to defective segmentation of daughter merozoites

**Fig 4:**
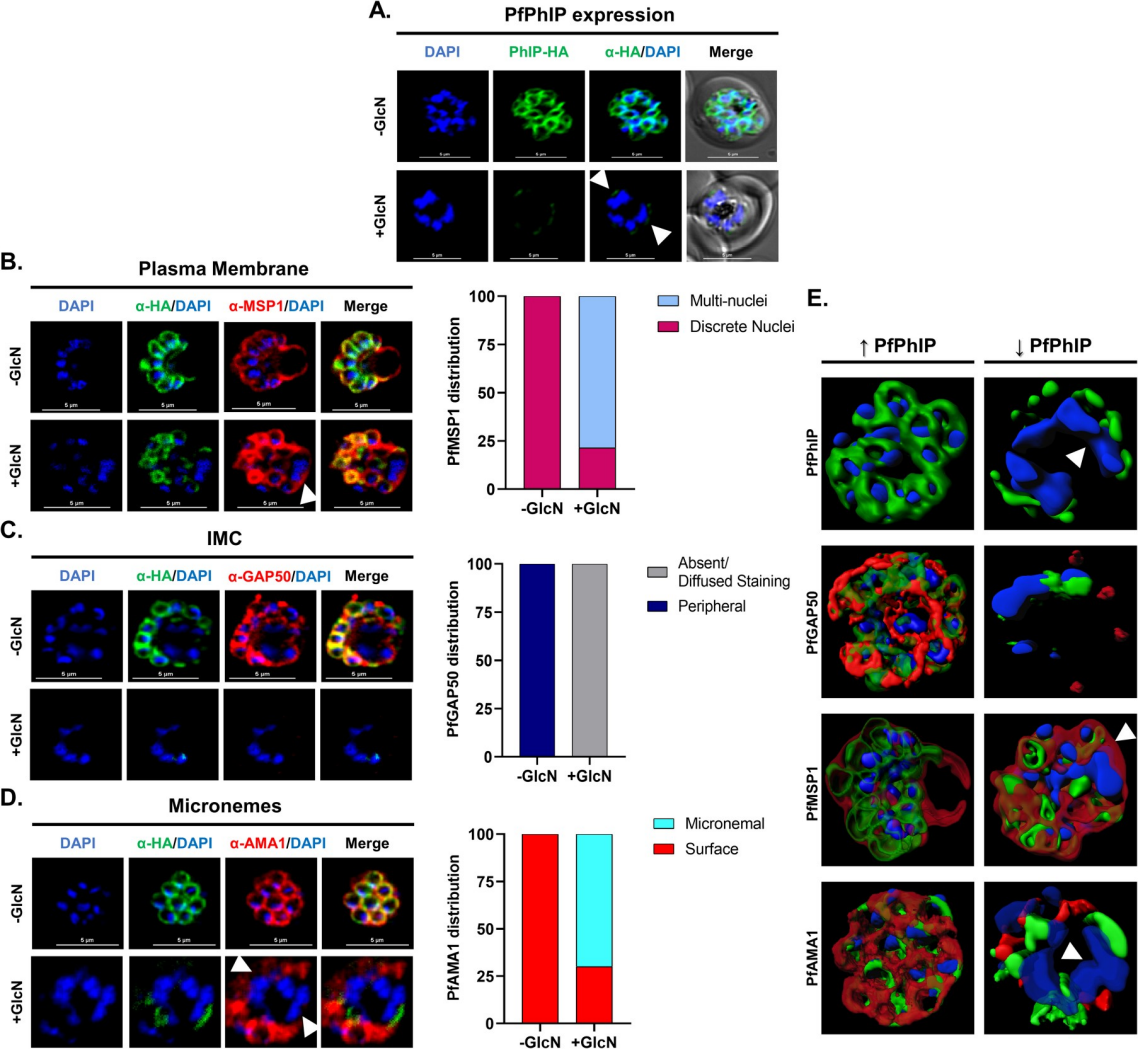
PfPhIP deficient parasites show defects in the segmentation of daughter cells. PfPhIP-HA-*glmS* parasites maintained with and without GlcN were E64-treated (10 μM) and probed with the anti-HA antibody (green) and counterstained with DAPI. **(A)** Apparent knockdown of PhIP expression leads to the formation of agglomerates of unsegmented daughter nuclei. **(B)** Anti-GAP50 antibody (IMC marker) (red) showed defects in IMC formation in agglomerates. Merozoite plasma membrane and micronemes were stained with antibodies against *P*. *falciparum* **(C)** merozoite surface protein 1 (PfMSP1) (red) and **(D)** PfAMA1 (red), respectively, in E64-treated schizonts cultivated with and without GlcN. PfMSP1 was visible in schizonts but forms relatively larger rings that surround multiple nuclei in the agglomerates. PfAMA1 showed a loss of signal in agglomerates. Scale bar = 5 μm. Based upon the staining pattern parasites were grouped and the percentage parasites in each group are presented in the bar graphs. **(E)** 3D reconstruction of the PfPhIP-HA-*glmS* schizonts with and without GlcN is presented using Imaris 7.6.1. Arrowheads indicate agglomerates of unsegmented daughter nuclei. See also S5 and S6 Figs.

We further dissected the defects in merozoite segmentation in PfPhIP knock-down parasites for the formation of the parasite plasma membrane (PPM), formation of and secretion by apical secretory organelles, and IMC formation using immunostaining. Briefly, schizonts maintained with and without GlcN from the early ring-stage were treated with 10 μM E64 at 42 hpi. E64 is a reversible protease inhibitor, which arrests the release of daughter merozoites from mature schizonts by preventing membrane proteolysis. In PhIP depleted parasites, multiple daughter cells remained partially attached to each other. In these parasites, segmented merozoites showed residual signal for PfPhIP, whereas in the unsegmented agglomerate PfPhIP staining was not detected (Fig 4A). PhIP depleted parasites showed apparent loss of signal for GAP50 in the multi-nucleated agglomerates suggesting a defect in IMC formation in agglomerates, while [–] GlcN parasites showed well-formed IMC around each nucleus of the segmented schizont (Fig 4B). Parasite plasma membrane which coats the individual newly formed daughter cells was examined by Merozoite Surface Protein 1 (MSP1). PhIP depleted schizonts showed MSP1 staining enclosing multiple nuclei of the agglomerate inside one contiguous membrane in contrast to untreated parasites that showed MSP1 surrounding each segmented daughter merozoite nucleus discretely (Figs 4C and S5A). Thus, in the agglomerates, PhIP knock-down parasites failed to direct the PPM around single daughter nuclei. Simultaneously microneme formation and secretion were assessed using anti-PfAMA1 (Apical Membrane Antigen 1) antibody in [–] GlcN as well as [+] GlcN parasites. Following the egress trigger, apical membrane antigen 1 (PfAMA1) is translocated from micronemes to the merozoite membrane. [–] GlcN PhIP-HA*glmS* parasites showed AMA1 staining around each merozoite whereas PhIP depleted parasites demonstrated surface AMA1 staining in fully segmented merozoites, while loss of AMA1 signal on merozoite surface was observed for the multi-nucleated agglomerates (Figs 4D and S5B). Quantitative analysis of immunofluorescence images for PhIP knockdown parasites w.r.t various marker antibodies was performed and plotted as percentage parasites showing respective staining patterns (Fig 4B, 4C and 4D). 3D reconstruction of schizont stage transgenic parasites with and without GlcN for different marker antibodies is illustrated using Imaris, version 7.6.1 (Fig 4E).

The ultra-structure of this incomplete budding in E64-treated schizonts with and without GlcN was examined by electron microscopy (S6 Fig). As expected, in the absence of GlcN, we observed distinct membrane-enclosed merozoites with organized rhoptries. In the presence of GlcN, only a few merozoites were separated from each other and a multi-nucleated agglomerate of unsegmented merozoites with randomly placed sets of organelles was present close to the food vacuole.

These results established that PfPhIP-deficient parasites show developmental defects during the final stages of schizont segmentation that fail to reinstate the asexual blood cycle due to structural defects.

### PfPhIP and PfGAPM2 knock-down results in the generation of non-invasive merozoites

**Fig 5:**
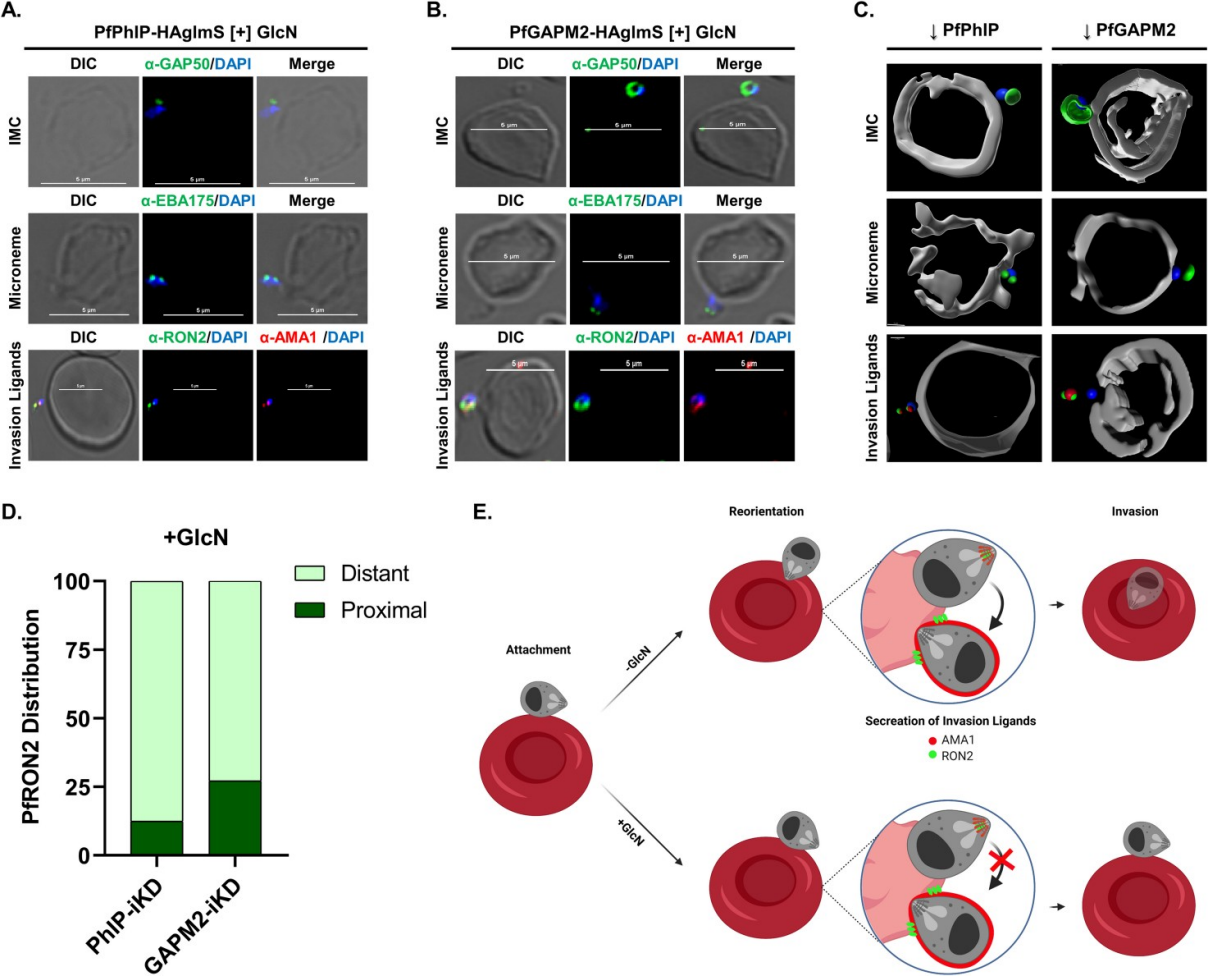
PfGAPM2 and PfPhIP function is essential for invasion of the host cell by the merozoites. Merozoites released from GlcN treated **(A)** PfPhIP-HA-glmS and **(B)** PfGAPM2-HA-glmS parasites were stained using antibodies against PfGAP50 (green, top panel), PfEBA175 (green, middle panel), PfRON2 (green) and PfAMA1 (red) (lower panel). Staining for these marker proteins showed normal micronemal and rhoptry organelles, however, it was observed that the discharge of contents from these apical organelles was affected. Also, the apical end of the merozoites, attached to the host was not aligned towards the erythrocyte membrane. Scale bar = 5 μm. **(C)** 3D reconstruction of the merozoites from PfPhIP or PfGAPM2 deficient parasites arrested on the RBC surface using Imaris 9. See also S7 Fig. **(D)** Percentage distribution of PhIP- and GAPM2-iKD parasites in the presence of glucosamine w.r.t to PfRON2 was quantified for staining pattern which is either distant from or in proximity to the erythrocyte surface. **(E)** Schematic showing the effect of PhIP and GAPM2 depletion on the secretion of invasion ligands from the apical complex of merozoite compared to a healthy, invasive merozoite.

Since we observed that the merozoites formed in PfPhIP and GAPM2 knock-down parasites were able to attach to the RBC surface, but could not invade, we next performed immunostaining of these RBC attached but non-invasive merozoites to determine whether the inability of merozoites to invade the RBCs is due to defects in apical organelles biogenesis or their secretion, which is crucial for the formation of the invasion complex; or due to the inability of motility complex which fails to propel the invading merozoite into the host RBC. To understand whether the IMC formation is affected in these knock-down parasites, we stained these attached merozoites with anti-PfGAP50 antibody. PhIP deficient merozoites showed loss of signal for GAP50 suggesting defects in IMC formation (Fig 5A; panel 1) while GAPM2 depleted merozoites displayed intact IMC encircling the nascent attached merozoite (Fig 5B; panel 1). To assess the merozoites released from the PhIP or GAPM2 deficient schizonts for the formation and secretion of apical organelles, we used EBA175, and AMA1 as markers for micronemes, and PfRON2 as a rhoptry marker. We observed typical micronemal staining as visualized by the presence of discrete dots with anti-EBA175 and anti-AMA1 antibodies. However, we could not locate these proteins on the merozoite surface in the merozoites released from the PhIP and GAPM2 knockdown schizonts, therefore indicating the failure to discharge the micronemal contents post egress (Fig 5A and 5B; panel 2 and 3). We additionally evaluated the presence of PfRON2, a rhoptry marker in merozoites in PfPhIP- and PfGAPM2-knockdown parasites. These knock-down parasites displayed characteristic rhoptry and micronemal localization (Fig 5A and 5B; panel 3). Following the egress, PfAMA1 is known to translocate from micronemes to the merozoite membrane and PfRON2 is inserted into the host membrane [13]. Together, these results indicated that merozoites formed and released in PfPhIP- and PfGAPM2-knockdown parasites were morphologically normal. However, apical organelle secretion of the invasion ligands seems to be affected in the merozoites released from schizonts with PfPhIP and PfGAPM2 deficiency (Figs 5A and 5B; panel 3 and S7A and S7B). 3D reconstruction of stuck merozoites from transgenic parasites in presence of GlcN with respect to different marker antibodies is illustrated using Imaris, version 7.6.1 (Fig 5C).

Since the secretion of EBA175, AMA1, and RON2, failed to initiate following merozoite attachment in PfPhIP- and PfGAPM2-deficient merozoites, it suggested that the signal for invasion in these merozoites post attachment to the erythrocytes was not triggered in PfPhIP- and PfGAPM2-knockdown parasites. On detailed analysis, we observed that ~87% and ~73% of the attached PhIP-iKD and GAPM2-iKD merozoites respectively, failed to align their apical end towards the erythrocyte surface i.e., the apex of the merozoite is not in direct proximity of the erythrocyte surface as indicated by staining with apical marker proteins (Fig 5D). Apical reorientation is imperative for triggering commitment to invasion. Taken together, the analysis of attached PfPhIP- and PfGAPM2-deficient merozoites suggested the role of PfPhIP and GAPM2 in the reorientation of merozoites, which is essential in positioning the apical organelles towards the erythrocyte membrane (Fig 5E). This study also highlights that merozoite reorientation might directly or indirectly be mediated by the motor complex due to the existence of the overlapping components among these two complexes.

## Discussion

A unifying feature of apicomplexan parasites is the presence of a surface pellicle and highly polarised cellular organization with the apical complex at the anterior end of their invasive stages, which is implicated in motility and invasion of host cells/tissue [14]. The IMC acts as an anchor for the motor complex, playing a significant role in gliding and thus invasion [5,15]. A number of studies have identified several proteins associated with the IMC and have provided insights into the organization and roles of the glideosome complex [5–7,16–18]. The basic motor complex in apicomplexan parasites that drives gliding motility and invasion is comprised of conserved components such as actin-MyoA-MTIP-GAP45-GAP40-GAP50-GAPMs-adhesin protein. The IMC has also been implicated to be involved in cell division [17].

Earlier, we and others have shown *Plasmodium* PhIL1 to be localized in parasite IMC and its association with alveolins and other protein components that overlap with those in the known glideosome complex, including GAP50 [10,11]. In the present study, we show the existence of PhIL1-associated complex and delineate the functional relevance of PhIL1-associated novel proteins in the *P*. *falciparum* merozoite namely, IMC1c or ALV5, a structural constituent of the SPN; a previously uncharacterized protein, PF3D7_1310700, which we termed PhIL1 Interacting Protein (PhIP); and GAPM2, a well-established component of the glideosomal complex. GFP-tagged proteins of PhIP, ALV5, and GAPM2 confirmed their localization to the IMC, and co-localization studies using indirect immunofluorescence assay affirmed the close association of these proteins with PhIL1 in the asexual blood stage of the parasite. The interactions between these proteins were also established by reciprocal co-precipitation studies using GFP-Trap wherein GFP tagged proteins from all the three parasite lines (ALV5-, PhIP- and GAPM2-GFP) were co-precipitated with their interacting protein partners and components of the PhIL1-associated complex.

Blue-native PAGE and co-sedimentation analysis of schizont stage parasite extracts confirmed the existence of PhIL1 and its associated proteins in complex(s). We could identify two discrete complexes; a low molecular mass complex corresponding to ~250kDa having PhIL1 and GAPM2 proteins and a high molecular weight complex of ~800kDa consisting of ALV5, PhIP, GAPM2, PhIL1, and GAP50, indicating the heterogeneity among these two complexes, which are composed of different but overlapping proteins. These results suggested a possible association of PhIL1-associated complex with the glideosomal complex. A far western assay using recombinant PhIL1 and GAP50 additionally confirmed the interaction between the two proteins and thus confirming GAP50 as a link between the two complexes. It is conceived that the motor complex assembles at the N-terminus of the GAP50 anchor [19]. It is possible that GAPM proteins, which span both the outer and inner sides of the IMC, may be part of different complexes on either side of the IMC. Therefore, we speculate that PhIL1 associates with GAP50 at the other end of the IMC i.e., at its C-terminus along with GAPMs and alveolins [20] and is a part of a novel complex at the inner IMC. Since GAP50 is both a luminal and transmembrane protein, it is possible that the PhIL1-associated complex interacts with GAP50 on the inner membrane of the IMC and GAP50 interacts on the outer side with the motor machinery proteins like GAP45, GAP40, MyoA, and MTIP [21–23].

We next characterized these selected components of the PhIL1-associated complex using conditional knock-down approaches. PfPhIP has been labelled as essential in the genome-wide screen in *P*. *falciparum* using random piggyBac transposon mutagenesis, while ALV5 and GAPM2 were scored as dispensable [24]. *Plasmodium* alveolins are thought to be involved in parasite motility through interactions with the pellicular membrane-embedded glideosomal components, apart from their role in morphogenesis and providing tensile strength. The conditional knockdown of PfALV5 slightly delayed the developmental time span at late stages of the asexual cycle, although the parasite could complete its growth leading to the formation of new rings. This could be due to the redundancy among 13 members of the alveolin family. These results are in line with the *Pb*IMC1d knock-out study where no apparent phenotype was observed [25]. Even a double knockout of alveolins (IMC1b and IMC1h) revealed decreased tensile strength of ookinetes without affecting their morphology [26]. It appears that glideosomal associated protein complexes exhibit considerable plasticity in their functions to ensure the survival of the parasite as suggested by various studies conducted in *T*. *gondii*. MyoA is reported to be recruited at the apical cap of the parasite through interactions with GAP45 or its paralog GAP70. Another member of this family, GAP80 recruits and assembles a new glideosome with MyoC at the basal polar region. Both these complexes share GAP50, GAP40, and MTIP. It was found that the deletion of MyoA is compensated by MyoC as it relocates to the apical end to initiate invasion and vice-versa [27]. These studies emphasize a high degree of complexity and functional versatility of the IMC components involved in gliding. Similar compensatory mechanisms have been reported for GAP45 and its ortholog at the basal region [15]. Recent investigations have demonstrated that parasites can invade the host cell in the absence of several core components of the glideosomal machinery (such as MyoA, MIC2, MLC1, GAP45, and actin), indicating the existence of alternative motor mechanisms for invasion [21–23]. All these studies uncover the fact that until now what was considered to be highly conserved machinery exhibits different protein complexes sharing some common components and displays complementary and compensatory mechanisms for successful invasion.

In contrast to the knock-down results for ALV5 parasites, PhIP and GAPM2 knock-down parasites showed a pronounced effect on parasite invasion. Following PhIP knockdown, a proportion of schizonts displayed incomplete segmentation and multiple merozoites remained attached to each other, while distinct merozoites were observed enclosed in schizonts in wild-type parasites as shown by light, immunofluorescence, and electron microscopy. This resultant phenotype might be due to the failure of IMC biogenesis and stabilization. A similar phenotype had been observed in the case of Merozoite Organising Protein (PfMOP) knock-down parasites, where agglomerates were noted due to flawed segmentation [28]. Also, this is in agreement with the PfSortilin knockdown study that showed the involvement of Sortilin in IMC biogenesis [29]. Together, the data suggested a possible role of PfPhIP in IMC formation in maturing schizonts, and failure to do so results in the absence of plasma membrane enclosing individual daughter merozoites in the agglomerates. Similar observations have been reported upon depletion of GAP40 and GAP50, which resulted in defective IMC biogenesis and stabilization during replication [16].

Some of the PhIP depleted schizonts were fully segmented and generated merozoites which egressed normally, however the merozoites released from these schizonts were unable to invade and got arrested on the surface of RBCs. These results suggest that despite the initial attachment, merozoites were unable to penetrate the host RBC, which might be due to impaired reorientation of the invading merozoites. The two distinct phenotypes observed due to the ablation of PhIP demonstrate the divergent functions of the components of IMC during the asexual lifecycle of the parasite. Recent work has provided evidence that specific proteins of the IMC can independently be involved in both motility and maintenance of cell shape and strength [26]. We speculate that PhIP might play a dual role in maintaining the cellular integrity of the daughter cells during cell division along with its role in the reorientation of merozoites during the invasion process.

The conditional knockdown of GAPM2-HA-glmS parasites showed the inability of merozoites to invade the RBCs. However, it was observed that the attachment of invasive merozoite is not affected in these parasites. While untreated GAPM2-HA-*glm*S parasites progressed to ring-stage infection. A recent study highlighted the role of GAPM1 and GAPM3 in providing the bridge between the sub-pellicular network and the alveoli in the IMC to maintain parasite structure and rigidity. GAPM1 depletion resulted in depolymerization of microtubules compromising parasite shape and integrity [30]. However, there has been no report suggesting the role of GAPMs in the invasion of the host.

Merozoite invasion is a complex, multistep process. First, there is a reversible attachment of merozoite to the erythrocyte surface through any part of the merozoite surface i.e., the apex of the merozoite is not in direct proximity of the erythrocyte surface followed by its apical reorientation so that the apical organelles are aligned to the erythrocyte membrane, formation of an irreversible tight junction (primarily involving AMA1 and RON) and ultimately its entry into the host cell powered through the motor complex. These steps are timed by organelle secretion and various signaling events [1]. To get insight into the defects in the invasion of merozoites released from PfPhIP and PfGAPM2 knockdown schizonts, we assessed the expression and secretion of the invasion ligands using EBA175, AMA1, and RON2. Post egress, AMA1 is exported from micronemes to the parasite surface and the secreted Rhoptry neck protein RON2 is inserted in the erythrocyte membrane, which binds to AMA1 on the merozoite surface and leads to the formation of a tight junction committing the merozoite to invade the host [13]. Despite showing an expected expression pattern for the apical organelles, the release of these invasion ligands onto the merozoite surface appeared to be affected. These defects in organelle secretion were found to be due to the failure of the attached merozoites to reorient their apical end towards the RBC surface, which in turn triggers the signaling events for the release of contents from apical organelles. The specific molecular interactions and mechanisms involved in apical reorientation are poorly understood. To date, there is no evidence for the involvement of the glideosome or its associated motor protein complexes in mediating the reorientation of apical organelles of the merozoites towards the host surface. A study (bioRxiv Preprint) recently identified the gliding ability of *Plasmodium* merozoites facilitated by actomyosin motor [31]. Data presented here suggest the role of PfPhIP and PfGAPM2 in the reorientation which might be directly or indirectly mediated through the motor complex in *P*. *falciparum* merozoites.

**Fig 6:**
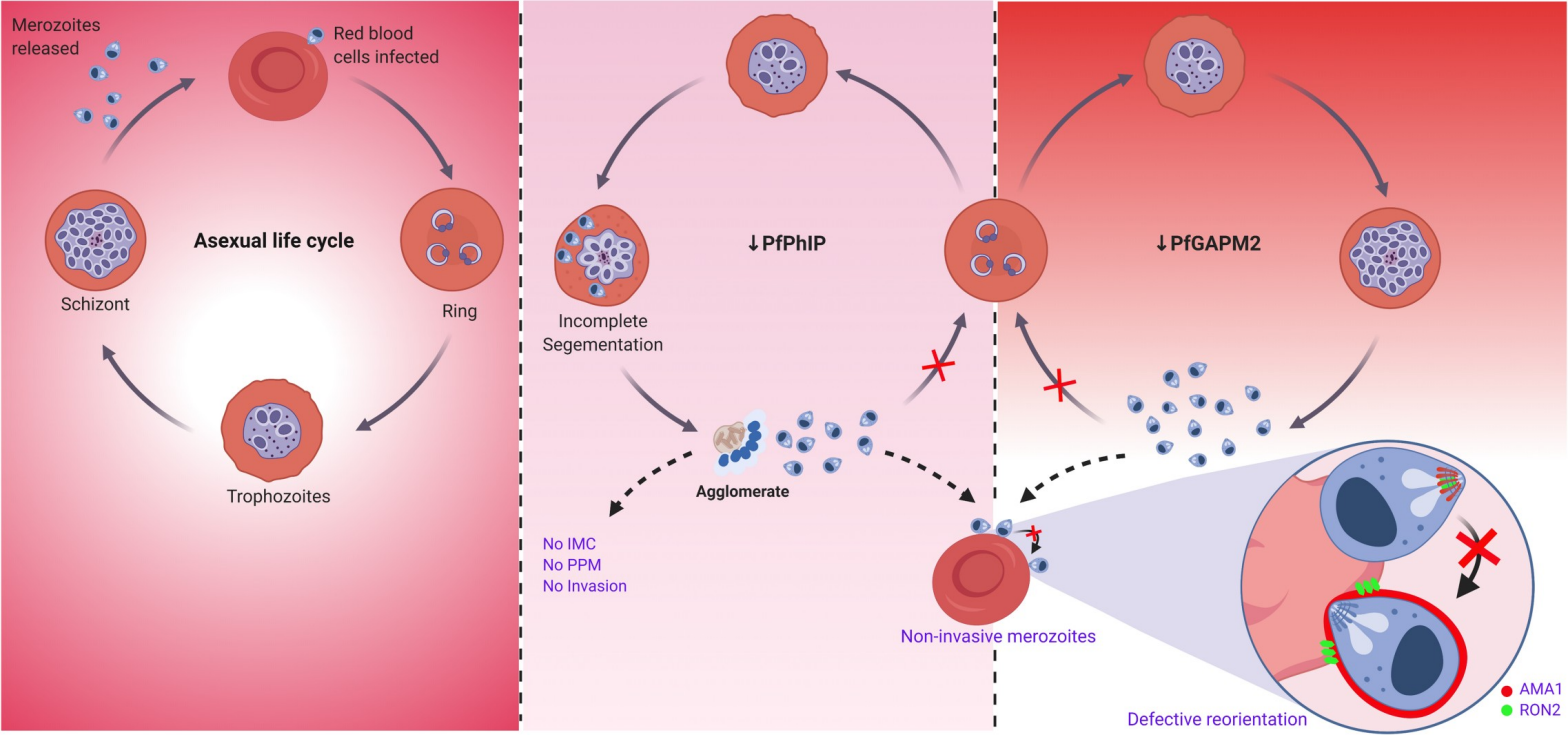
Function of PhIL1-associated complex is crucial for reorientation mediated by gliding motor complex and thus host-cell invasion by *Plasmodium falciparum* merozoites. The schematic illustrates GAPM2 and PhIP are essential for blood-stage infection and their genetic attenuation arrests merozoite invasion by impeding the function of glideosomal motor machinery resulting in failure of merozoite to reorient its apical end towards the host RBC. Depletion of PhIP also leads to the formation of agglomerates of unsegmented schizonts suggesting its probable role in IMC-mediated parasite cell division. In the left panel, the level of PfPhIP or GAPM2 protein is maintained by the absence of GlcN. The middle panel shows consequences of PhIP deficiency, wherein a portion of daughter cells get segmented while others remain trapped as an agglomerate under a common plasma membrane. In the agglomerate, IMC fails to form, parasite plasma membrane surrounds multinucleated unsegmented daughter cell, and microneme secretion is affected. Few segmented merozoites which egressed were seen arrested at the erythrocyte surface and are unable to invade. The right panel summarizes the effect of GAPM2 deficiency in the parasite where the merozoites fail to invade the erythrocytes due to the disability of these merozoites to align their apical end towards the host cell surface. Apical reorientation of merozoites is imperative for invasion so that the apical organelles are aligned to the erythrocyte membrane which leads to the discharge of apical organellar proteins followed by invasion. Created with BioRender.com.

In conclusion, we have characterized *P*. *falciparum* ALV5, PhIP, a previously uncharacterized protein, and GAPM2 and show that GAPM2 and PhIP are essential for the blood-stage infection as their genetic attenuation arrests merozoite invasion resulting from the failure of merozoite to reorient its apical end towards the host RBC (Fig 6). Taken together, our results suggest that the PhIL1-associated IMC complex is different in composition from that of the previously described glideosomal complex and it appears to be essential for parasite invasion into host erythrocytes. The study thus provides new molecular and mechanistic insights into the contribution of IMC which will likely be useful to identify new molecules for intervention strategies for malaria parasite development.

### Experimental procedures

#### Maintenance of *P*. *falciparum* cultures

*P*. *falciparum* parasite line 3D7 was maintained in O^+^ human erythrocytes (RBC) at 3% hematocrit in RPMI 1640 medium (pH 7.4) supplemented with 50 μg/ml hypoxanthine, 0.5% albumax II, 2 mg/ml sodium bicarbonate, and 20 μg/ml gentamycin. Cultures were incubated in airtight boxes at 37°C in an atmosphere of 1% O_2_, 4% CO_2_ and 95% N_2_ [32]. Parasites were synchronized using sorbitol treatment [33]. Briefly, the culture was harvested at about 10% parasitemia with a majority of parasites at the ring stage by centrifuging at 2000 rpm for 5 min at RT. To the cell pellet, 5 volumes of 5% sorbitol solution was added and mixed gently. This solution was incubated at 37°C for 10 min and centrifuged at 2000 rpm for 5 min. The supernatant was carefully discarded without disturbing the pellet. Pellet was washed with prewarmed complete media twice and the culture was incubated at 37°C for the growth of the parasite.

#### *P*. *falciparum* parasite transfection

To generate a GFP-tagged transfection vector construct, the entire open reading frame of PfPhIP, PfALV5, and PfGAPM2 were amplified using gene-specific primers (S1 Table) and cloned into the transfection vector pSSPF2 [34] at the *Bgl*II and *Avr*II restriction sites to create a fusion of the desired gene of interest (GOI) with GFP under the control of the *hsp86* promoter. Synchronized *P*. *falciparum* 3D7 ring-stage parasites were transfected with 100 μg of purified plasmid DNA by electroporation (310 V, 950 μF) and the transfected parasites were selected using 2.5 nM blasticidin [35]. To detect expression of the PfGOI-GFP fusion protein in the transgenic line, parasite lysates were analyzed with western blotting using anti-GFP antibody.

For the generation of knock-down constructs, the C-terminal region of PfPhIP, PfALV5, and PfGAPM2 was amplified using gene-specific primers (S1 Table) and cloned into the transfection vector pHA-glmS [36] using *Pst*I and *Bgl*II restriction sites to create a fusion of the desired gene of interest (GOI) with HA-glmS under the control of the native promoter. The ring-stage parasites were transfected as mentioned and transgenic parasites were selected on alternate blasticidin drug ON and OFF cycles to ensure genomic integration of PfGOI-HA-glmS constructs. The transgenic parasites were then subjected to clonal selection by serial dilution to obtain a parasite line from a single genome integrated clone.

#### Isolation of parasites, extraction of proteins, and immunoblotting

Expression of the PfGOI-GFP or PfGOI-HA fusion protein in transgenic *P*. *falciparum* blood-stage parasites was examined by western blotting as described previously [11]. Briefly, schizont stage parasites were isolated following lysis of infected erythrocytes with 0.15% saponin; following centrifugation the pellet was resuspended in RIPA buffer (150 mM NaCl, 1% Nonidet P-40, 0.5% sodium deoxycholate, 0.1% SDS, 50 mM Tris pH 8.0) and cells were lysed at 4°C for 30 min, followed by 3 cycles of freeze-thaw in liquid N_2_ and at 37°C. A clear parasite lysate was obtained by centrifugation at 13,000 rpm for 30 min at 4°C. The supernatant was then mixed with Laemmli buffer, boiled, and centrifuged, and proteins were separated on 12% SDS-PAGE. The fractionated proteins were transferred from the gel onto the PVDF membrane (Millipore) for 2 h at 200 mA and then the membrane was treated with blocking buffer (5% milk powder in 1 × PBS) overnight at 4°C. The blot was incubated for 1 h with rabbit anti-GFP (1: 10,000) or rat anti-HA (1:1000), followed with secondary anti-rabbit/anti-rat IgG antibody (1: 300,000) conjugated to HRP. Protein bands were visualized using an ECL detection kit (Thermo Scientific, USA).

#### Fluorescence microscopy

To visualize GFP expression, the transgenic parasite suspension was incubated with DAPI (2 ng/ml) in PBS at RT for 10 min. Following three washes with 1 × PBS (pH 7.4); samples were mounted on glass slides and observed on a Nikon A1 Confocal Microscope (Nikon Corporation, Tokyo, Japan).

#### Indirect immunofluorescence assay

Parasites were fixed with fixation solution containing 4% paraformaldehyde and 0.0075% glutaraldehyde in PBS for 30 min and subjected to permeabilization with 0.1% Triton X-100 followed by blocking with 10% FBS. Parasites were then probed with primary antibody for 1 h, followed by the secondary antibody for 1 h at RT. The parasites were incubated with DAPI to stain the nucleus for 10 min at RT and imaged using a Nikon A1-R confocal microscope (Nikon Corporation, Tokyo, Japan). The images were analyzed by NIS elements software (Nikon). The antibody combinations used for various experiments are 1. For colocalization with the GFP line, rabbit α-PfPhIL1 (1:100) was used, 2. For the colocalization studies in knockdown experiments, rabbit α-PfGAP50 (1:100), rabbit anti-PfMSP1 (1:250), rabbit α-PfAMA1 (1:100), rabbit α-PfEBA175 (1:100), mice α-PfRON2 (1:50) and rat α-HA (1:100) were used; followed by appropriate secondary antibodies: anti-mice Alexafluor 488 (1:500), anti-rabbit Alexafluor 488 (1:500), anti-rabbit Alexafluor 594 (1:500), and anti-rat Alexafluor 488 (1:500) (Invitrogen).

#### Conditional knock-down assay

The functional role of PfALV5, PfPhIP, and PfGAPM2 was determined by inducible knockdown with glucosamine. Glucosamine was added to the parasite lines (PfGOI-HA-glmS transgenic and 3D7) with hematocrit and parasitemia of synchronized ring-stage culture adjusted to 2% and 1%, respectively, at varying concentrations (0.3 mM, 0.6 mM, 1.25 mM, 2.5 mM, and 5 mM). Parasite growth was monitored microscopically after every 8 h by Giemsa-stained smears. The parasitemia was estimated after an incubation of 40 h in the next cycle and percent inhibition was calculated relative to the GlcN untreated PfGOI-HA-glmS parasite cultures. Briefly, cells from samples were collected and washed with PBS followed by staining with ethidium bromide (10 μg/ml) for 20 min at 37°C. The cells were subsequently washed twice with PBS and analyzed on FACSCalibur (Becton Dickinson) using the Cell Quest Pro software. Fluorescence signal (FL2) was detected with the 590 nm bandpass filter using an excitation laser of 488nm collecting 100000 cells per sample. Uninfected RBCs stained similarly were used as a control.

#### Co-precipitation of interacting proteins

For Pull-down of GFP-fusion proteins, schizont stage lysate was obtained as described above and immunoprecipitation was done using GFP-Trap_A Kit (Chromotek) following the manufacturer’s instructions. GFP-Trap_A beads were equilibrated with dilution buffer and allowed to bind to proteins in the parasite lysate by tumbling the tube end-over-end for 3 h at 4°C. Samples were then centrifuged at 1600 rpm for 1 min and the beads were washed twice with dilution buffer. Proteins were eluted in 50 μl elution buffer and peptides were analyzed by mass spectrometry.

#### Glycerol density gradient fractionation for isolation of complexes

The sedimentation curve of molecular mass standards in different fractions and the protocol followed are as described earlier [37]. Briefly, schizont stage parasites were lysed as described above. The lysate was cleared by centrifugation, and 500 μL of lysate was layered on top of a 9 mL 5–45% glycerol step-gradient (45% glycerol solution being the lowermost and 5% glycerol solution being the topmost layer). Gradients were centrifuged at 38,000 rpm for 18 h at 4°C in an SW41 Ti rotor (Beckman). Twenty, 0.5 mL fractions were collected from each gradient, and equal volumes of each fraction were mixed with sample loading dye. Alternate protein fractions were resolved by SDS/PAGE and analyzed by Western blotting using protein-specific antibodies at 1:5000 dilution for GAP50, PhIL1, ALV5, PhIP and GAPM2 followed by secondary α-rabbit antibody at 1:100000 dilution and detection using an ECL detection kit (Thermo Scientific, USA).

#### Native gel electrophoresis

Native-PAGE analysis was carried out to separate various complexes present in the parasite lysate using NativePAGE Novex Bis-Tris Gel System (Life Technologies) following the manufacturer’s protocol. Briefly, Schizont stage parasite lysate was mixed with NativePAGE sample buffer and NativePAGE 5% G-250 sample additive and resolved in the NativePAGE Novex 4–16% Bis-Tris Gels at 4°C, which resolve proteins in the molecular weight range of 15–1,000 kDa and then transferred to polyvinylidene difluoride (PVDF) membranes. To identify PfPhIL1-associated complexes, the membrane was probed with rabbit anti-PfGAP50, anti-PfPhIL1, anti-PfALV5, anti-PfPhIP, and anti-PfGAPM2 (1:5000 dilution each) antisera, followed by incubation with HRP-conjugated goat anti-rabbit IgG secondary antibody (1:100,000).

#### Transmission electron microscopy

PfPhIP-HAglmS parasites [+]/ [–] GlcN were percoll purified at 42 hpi and treated with 10 μM E64. Parasites were washed in phosphate buffer (pH 7.0) and fixed with 1% paraformaldehyde. [–] GlcN parasites were fixed at 48 hpi and [+] GlcN parasites were fixed at 52 hpi. Parasites were then resuspended in 2.5% glutaraldehyde followed by the addition of 2% Osmium Tetroxide and washed with phosphate buffer. The samples were then subjected to subsequent dehydration in grades of alcohol (50%, 60%, 70%, 80%, 90%, and 100%). The samples were then infiltrated in Epoxy resin composed of ERL-4221, DER 736, DMAE, and NSA (Ted Pella, Inc). The following day, samples were embedded in Epoxy resin and baked at 65°C for 16h. Ultrathin sections (about 90nm) were cut on ultramicrotome RMC Boeckeler PTPC, samples were stained using lead citrate followed by 1% Uranyl acetate, visualized in Tecnai G2 spirit (FEI, Netherland), operating at a voltage of 120kV, and images were recorded with a Mega View III (SIS, Germany) digital camera.

#### Expression and purification of PfGAP50

GAP50-pET 28a construct was used for expression in Rosetta2 pLysS system in LB medium containing kanamycin (50 μg/ml) and chloramphenicol (34 μg/ml) at 37°C with 6 μM CoSo_4_. Cells were induced at 18°C with 0.3 mM isopropyl β-D thiogalactopyranoside (IPTG) for 16 hrs. When OD_600_ reached 2.0, the cell suspension was pelleted down (1500g, 20 min, 4°C) and cells were resuspended in 50 mM Tris pH 7.4, 300 mM NaCl, 10 mM imidazole, and 10% glycerol (Buffer A). Cells were lysed by sonication and lysate was centrifuged (45 min, 15000g, 4°C). The supernatant was passed through Ni-NTA resin pre-equilibrated buffer A at 4°C. After binding, 30 CV wash was given with buffer A and protein was eluted with 500 mM imidazole in buffer A.

#### Far western blotting

The far western assay was carried out according to the protocol described by Xing-Zhen Chen and colleagues [12].

## Supporting information

S1 Fig Generation and conformation of PfALV5-, PfPhIP- and PfGAPM2-GFP fusion parasite lines. (A) Schematic showing vector map of pSSPF2 construct, indicating different vector cassettes used for generation of GFP protein in fusion with PfALV5, PfPhIP, or PfGAPM2. Western blot analysis of lysate from (B) PfALV5-GFP, (C) PfPhIP-GFP, and (D) PfGAPM2-GFP tag line, with α-GFP rabbit serum. M denotes known molecular weight marker.
(TIF)

S2 Fig Glycerol gradient fractionation for cosedimentation of PhIL1-associated complex along with PfGAP50. Western blot analysis following glycerol gradient centrifugation in *P. falciparum* blood-stage schizonts using protein-specific antibodies for (A) PfGAP50 (B) PfPhIL1 (C) PfALV5 (D) PfPhIP and (E) PfGAPM2. n = 2 experiments. M denotes known molecular weight marker.
(TIF)

S3 Fig Generation of PfALV5, PfPhIP, and PfGAPM2 inducible knockdown parasite lines. (A) Schematic of the glmS ribozyme reverse genetic tool: The ribozyme is inserted in the 3′-UTR after the coding region so that it is present in the expressed mRNA. Following the addition of the inducer, glucosamine, which binds to the ribozyme, the mRNA self-cleaves resulting in degradation of the mRNA and knockdown of protein expression. Integration into the parasite genome was confirmed by PCR using different primer sets: cloned C-terminus region (a/b), upstream of the cloned region (c), and from the glmS ribozyme sequence, 1236A (d). The position of primers is marked by arrowheads. (B) PfALV5-pHA_glmS integrants in parasite genome were selected by PCR analysis using primer sets: 1003600_FglmS-HA (a) / 1003600_RglmS-HA (b) and 1003600_Int. (c) /1236A (d). (C) PCR was set up for confirmation of successful integration of PfPhIP-pHA_glmS construct in parasite genome using different set of primers: 1310700_FglmS-HA (a) /1310700_RglmS-HA (b) and 1310700_Int (c) /1236A (d). (D) Successful integration of PfGAPM2-pHA_glmS construct in parasite genome was confirmed using primer set: 0423500_FglmS-HA (a) / 0423500_RglmS-HA (b) and 0423500_Int. (c) / 1236A (d). M denotes known molecular weight marker.
(TIF)

S4 Fig The two distinct phenotypes observed in PhIP knock-down parasites demonstrate the divergent functions of the components of IMC during the asexual lifecycle of the parasite. **(A)** The number of parasites showing the arrest in development following knockdown due to two different phenotypes was calculated from Giemsa-stained smears of PhIP-HA-*glmS* parasites after 42 h of glucosamine treatment (1.25 mM). **(B)** Representative parasites from the Giemsa-stained smears showing agglomerates and arrested merozoites following PfPhIP knockdown.
(TIF)

S5 Fig PfPhIP deficiency leads to incomplete formation of the IMC. Representative images of E64-treated schizont stage in [–]/ [+] GlcN PfPhIP-HA-*glmS* parasites using antibodies against **(A)** PfMSP1 and **(B)** PfAMA1. Arrowheads show agglomerates showing loss of signal for AMA1 and MSP1 in unsegmented nuclei. Scale bar = 5 μm.
(TIF)

S6 Fig PfPhIP knock-down causes developmental defects during schizogony. Transmission electron micrographs of PfPhIP-HAglmS parasites [–] and [+] GlcN. Arrowheads in PfPhIP-deficient schizonts point to incompletely segmented daughter cells (red) and agglomerates of multiple daughter nuclei (green), while distinct membrane-enclosed merozoites with well-arranged apical organelles were observed in GlcN untreated schizonts. Scale bars = 2000 nm.
(TIF)

S7 Fig Released merozoites from PfPhIP- and PfGAPM2-deficient schizonts fail to invade erythrocytes. Merozoites appeared to be arrested at RBC surface due to failure to align its apical end towards the host cell were probed with DAPI; anti-RON2 (green), anti-GAP50 (green), anti-EBA175 antibody (green) and anti-AMA1 antibody (red) in **(A)** PfPhIP deficient merozoites **(B)** PfGAPM2 deficient merozoites. Scale bar = 5 μm.
(TIF)

S1 Table Oligonucleotides used in this study. (TIF)
