# Supplementary figures and images for: Plasmodium falciparum PhIL1-associated complex plays an essential role in merozoite reorientation and invasion of host erythrocytes
Source: PLoS Pathog. 2021 Jul 29;17(7):e1009750. doi: 10.1371/journal.ppat.1009750 (PMC8321122; doi:10.1371/journal.ppat.1009750)

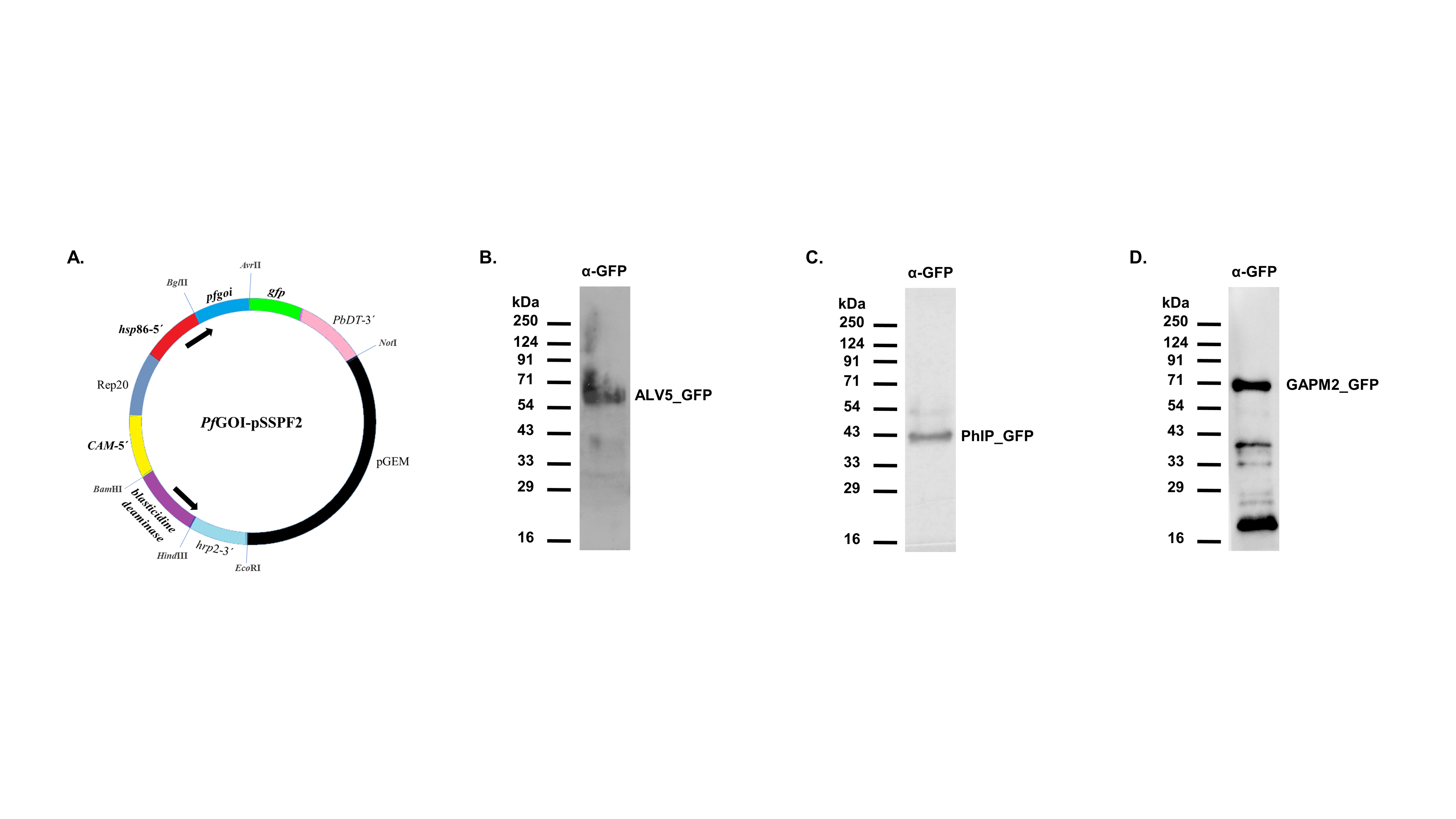

Supplement: S1 Fig — (A) Schematic showing vector map of pSSPF2 construct, indicating different vector cassettes used for generation of GFP protein in fusion with PfALV5, PfPhIP, or PfGAPM2. Western blot analysis of lysate from (B) PfALV5-GFP, (C) PfPhIP-GFP, and (D) PfGAPM2-GFP tag line, with α-GFP rabbit serum. M denotes known molecular weight marker. (TIF) [file ppat.1009750.s001.tif]

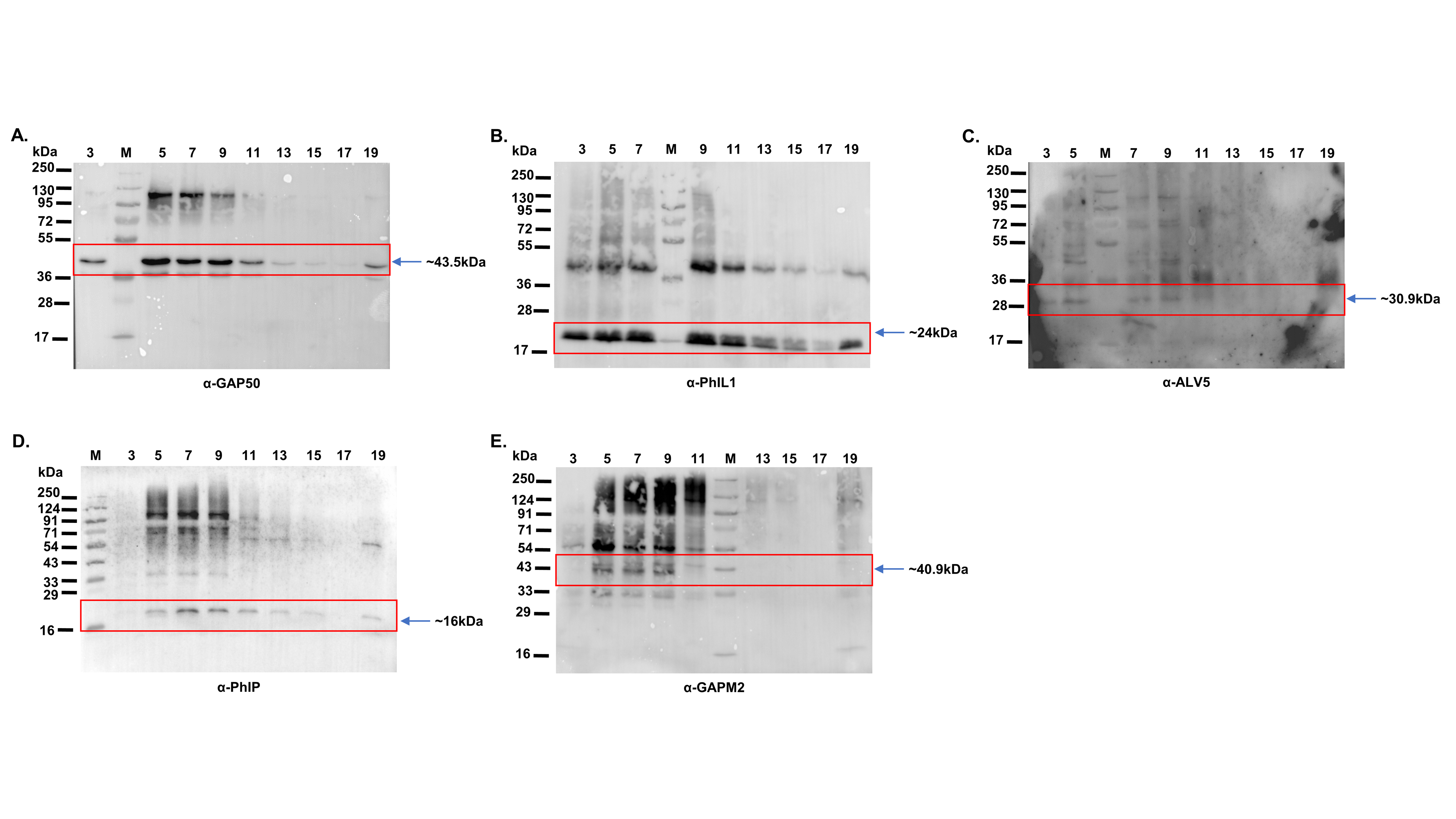

Supplement: S2 Fig — Western blot analysis following glycerol gradient centrifugation in P. falciparum blood-stage schizonts using protein-specific antibodies for (A) PfGAP50 (B) PfPhIL1 (C) PfALV5 (D) PfPhIP and (E) PfGAPM2. n = 2 experiments. M denotes known molecular weight marker. (TIF) [file ppat.1009750.s002.tif]

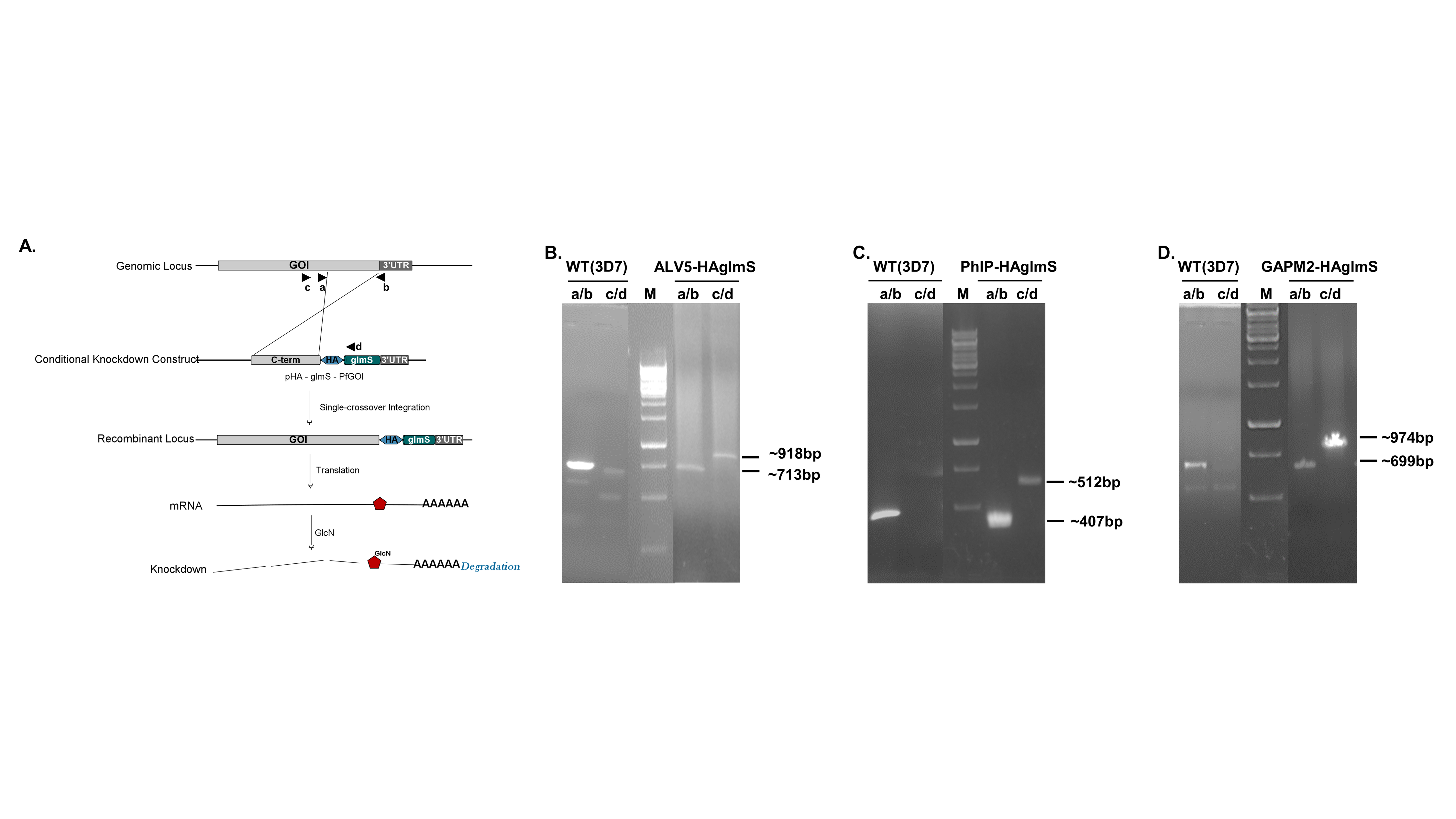

Supplement: S3 Fig — (A) Schematic of the glmS ribozyme reverse genetic tool: The ribozyme is inserted in the 3′-UTR after the coding region so that it is present in the expressed mRNA. Following the addition of the inducer, glucosamine, which binds to the ribozyme, the mRNA self-cleaves resulting in degradation of the mRNA and knockdown of protein expression. Integration into the parasite genome was confirmed by PCR using different primer sets: cloned C-terminus region (a/b), upstream of the cloned region (c), and from the glmS ribozyme sequence, 1236A (d). The position of primers is marked by arrowheads. (B) PfALV5-pHA_glmS integrants in parasite genome were selected by PCR analysis using primer sets: 1003600_FglmS-HA (a) / 1003600_RglmS-HA (b) and 1003600_Int. (c) /1236A (d). (C) PCR was set up for confirmation of successful integration of PfPhIP-pHA_glmS construct in parasite genome using different set of primers: 1310700_FglmS-HA (a) /1310700_RglmS-HA (b) and 1310700_Int (c) /1236A (d). (D) Successful integration of PfGAPM2-pHA_glmS construct in parasite genome was confirmed using primer set: 0423500_FglmS-HA (a) / 0423500_RglmS-HA (b) and 0423500_Int. (c) / 1236A (d). M denotes known molecular weight marker. (TIF) [file ppat.1009750.s003.tif]

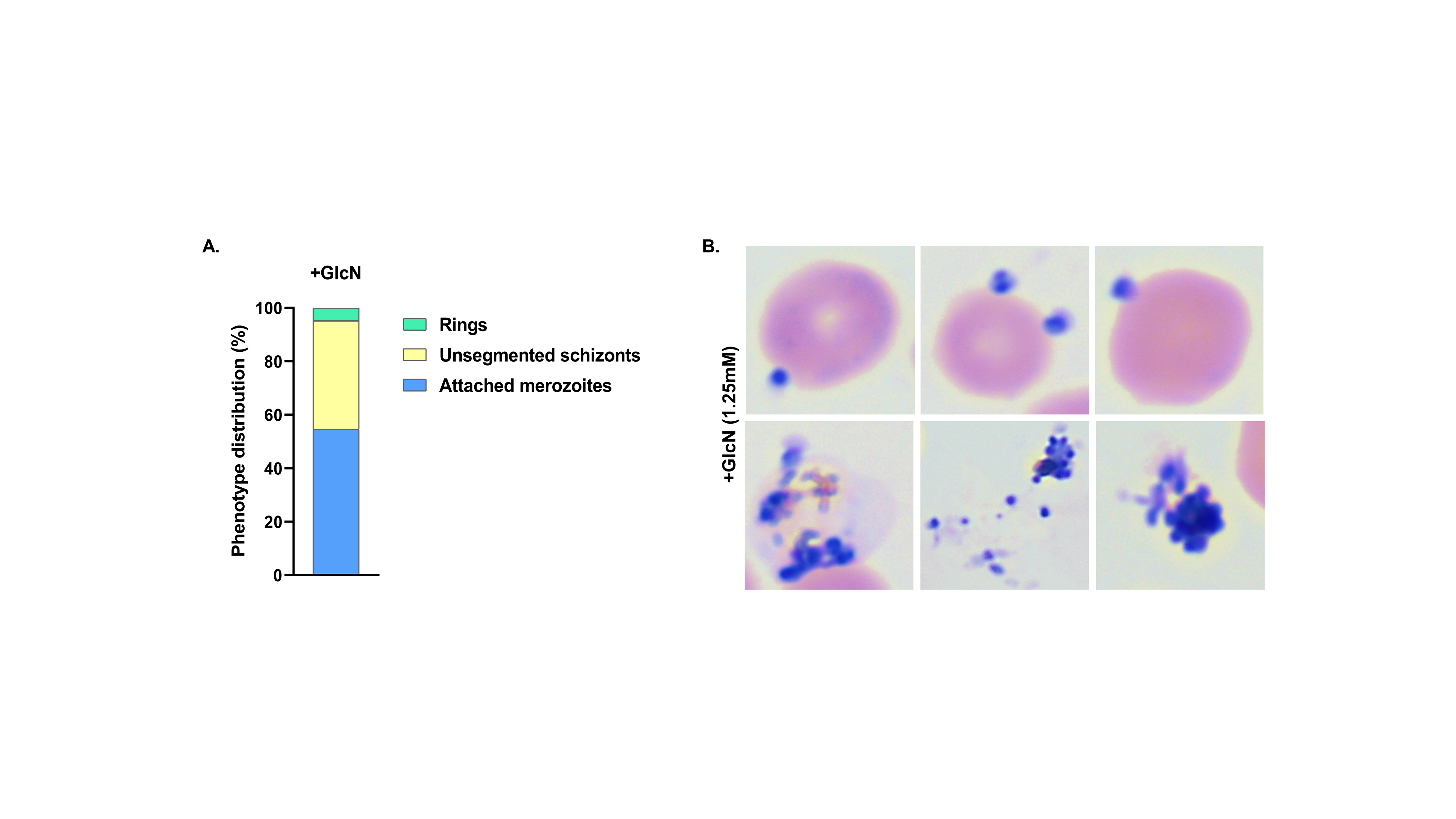

Supplement: S4 Fig — (A) The number of parasites showing the arrest in development following knockdown due to two different phenotypes was calculated from Giemsa-stained smears of PhIP-HA-glmS parasites after 42 h of glucosamine treatment (1.25 mM). (B) Representative parasites from the Giemsa-stained smears showing agglomerates and arrested merozoites following PfPhIP knockdown. (TIF) [file ppat.1009750.s004.tif]

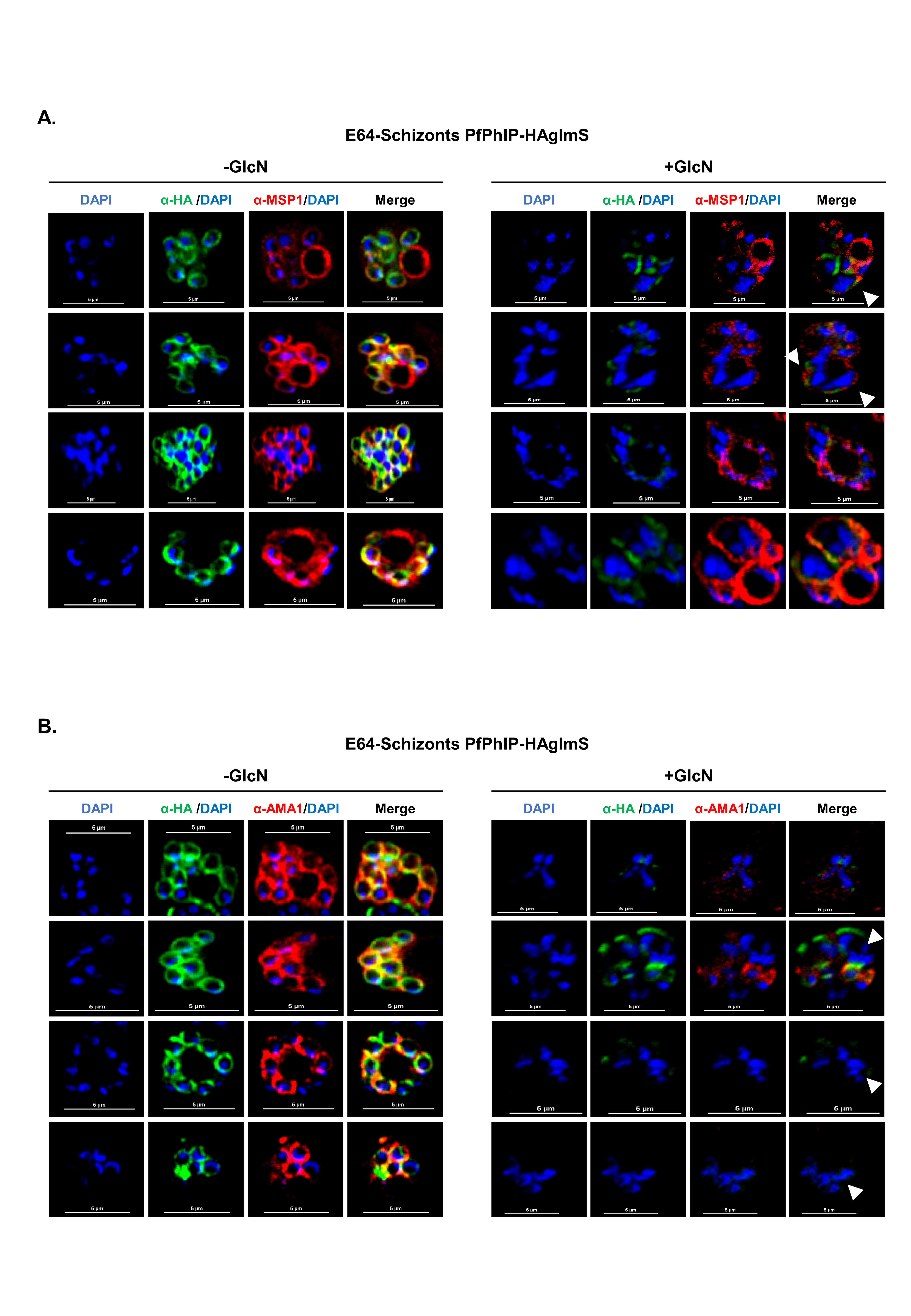

Supplement: S5 Fig — Representative images of E64-treated schizont stage in [–]/ [+] GlcN PfPhIP-HA-glmS parasites using antibodies against (A) PfMSP1 and (B) PfAMA1. Arrowheads show agglomerates showing loss of signal for AMA1 and MSP1 in unsegmented nuclei. Scale bar = 5 μm. (TIF) [file ppat.1009750.s005.tif]

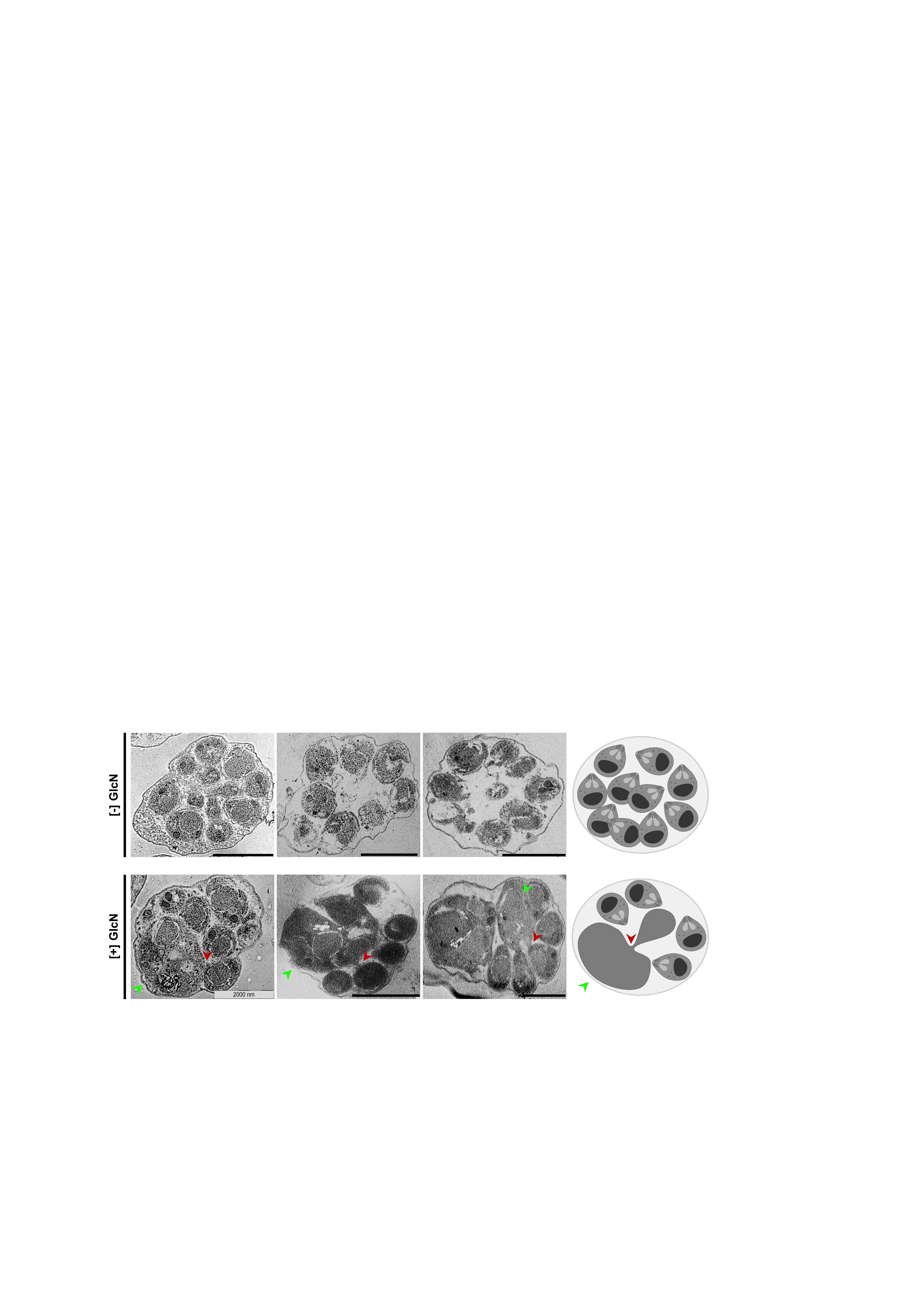

Supplement: S6 Fig — Transmission electron micrographs of PfPhIP-HAglmS parasites [–] and [+] GlcN. Arrowheads in PfPhIP-deficient schizonts point to incompletely segmented daughter cells (red) and agglomerates of multiple daughter nuclei (green), while distinct membrane-enclosed merozoites with well-arranged apical organelles were observed in GlcN untreated schizonts. Scale bars = 2000 nm. (TIF) [file ppat.1009750.s006.tif]

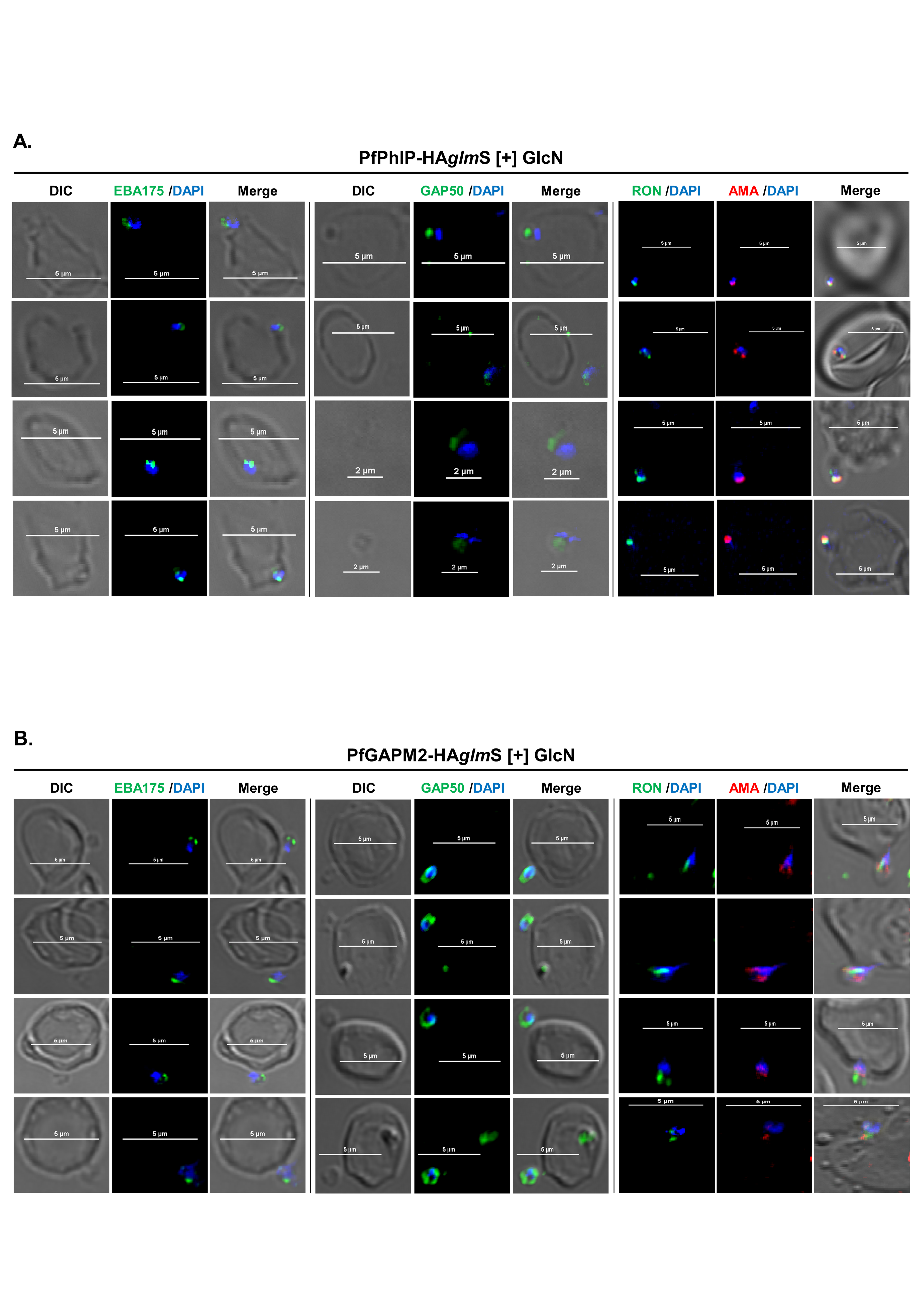

Supplement: S7 Fig — Merozoites appeared to be arrested at RBC surface due to failure to align its apical end towards the host cell were probed with DAPI; anti-RON2 (green), anti-GAP50 (green), anti-EBA175 antibody (green) and anti-AMA1 antibody (red) in (A) PfPhIP deficient merozoites (B) PfGAPM2 deficient merozoites. Scale bar = 5 μm. (TIF) [file ppat.1009750.s007.tif]

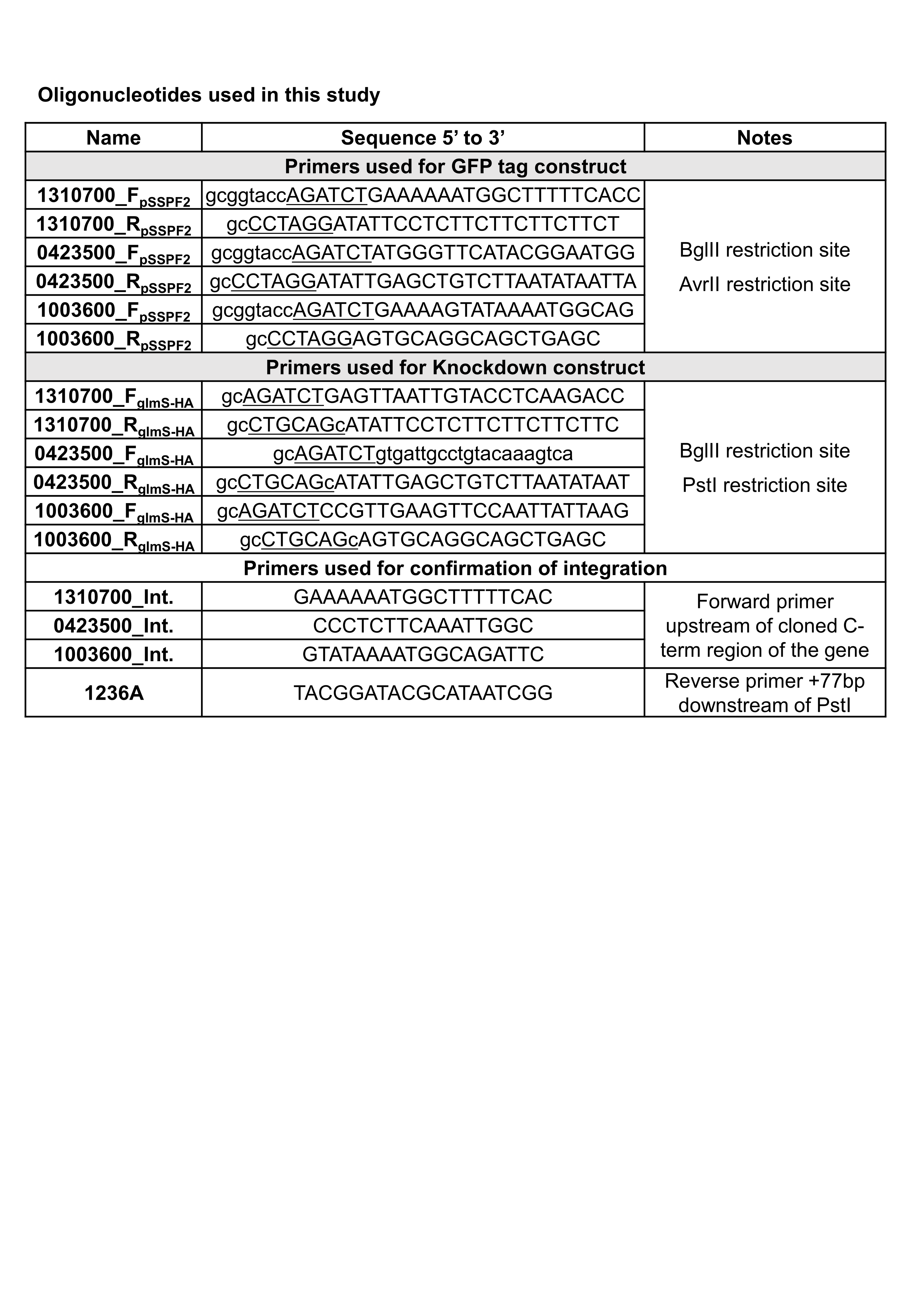

Supplement: S1 Table — (TIF) [file ppat.1009750.s008.tif]
